# An Approach to Developing Cyanines with Upconverted Photosensitive Efficiency Enhancement for Highly Efficient NIR Tumor Phototheranostics

**DOI:** 10.1002/advs.202202885

**Published:** 2022-09-12

**Authors:** Xueze Zhao, Shan He, Weijie Chi, Xiaogang Liu, Pengzhong Chen, Wen Sun, Jianjun Du, Jiangli Fan, Xiaojun Peng

**Affiliations:** ^1^ State Key Laboratory of Fine Chemicals Frontiers Science Center for Smart Materials Oriented Chemical Engineering Dalian University of Technology Dalian 116024 P. R. China; ^2^ State Key Laboratory of Molecular Reaction Dynamics and Dynamics Research Center for Energy and Environmental Materials Dalian Institute of Chemical Physics Chinese Academy of Sciences Dalian 116023 P. R. China; ^3^ Fluorescence Research Group Singapore University of Technology and Design Singapore 487372 Singapore; ^4^ Ningbo Institute of Dalian University of Technology Ningbo 315016 P. R. China; ^5^ State Key Laboratory of Fine Chemicals, College of Materials Science and Engineering Shenzhen University Shenzhen 518057 P. R. China

**Keywords:** cyanine dyes, heavy‐atom‐free, near‐infrared, one‐photon upconversion, photosensitization

## Abstract

Upconverted reactive oxygen species (ROS) photosensitization with one‐photon excitation mode is a promising tactic to elongate the excitation wavelengths of photosensitive dyes to near‐infrared (NIR) light region without the requirement of coherent high‐intensity light sources. However, the photosensitization efficiencies are still finite by the unilateral improvement of excited‐state intersystem crossing (ISC) via heavy‐atom‐effect, since the upconverted efficiency also plays a decisive role in upconverted photosensitization. Herein, a NIR light initiated one‐photon upconversion heavy‐atom‐free small molecule system is reported. The meso‐rotatable anthracene in pentamethine cyanine (Cy5) is demonstrated to enrich the populations in high vibrational–rotational energy levels and subsequently improve the hot‐band absorption (HBA) efficiency. Moreover, the spin–orbit charge transfer intersystem crossing (SOCT‐ISC) caused by electron donated anthracene can further amplify the triplet yield. Benefiting from the above two aspects, the ^1^O_2_ generation significantly increases with over 2‐fold improved performance compared with heavy‐atom‐modified method under upconverted light excitation, which obtains efficient in vivo phototheranostic results and provides new opportunities for other applications such as photocatalysis and fine chemical synthesis.

## Graphical abstract

A heavy‐atom‐free molecular design approach for achieving NIR upconverted photosensitization is reported. By modifying rotatable electron donating group at the *meso* position of Cy5, both the hot band absorption efficiency and intersystem crossing are improved. Moreover, the instructive design approach is also applicable in Cy5.5 system compounds, which provides new opportunities for NIR photosensitization applications.

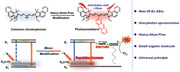

## 1 Introduction

Near‐infrared light mediated reactive oxygen species (ROS) generation and luminescence play crucial roles in diverse areas, such as photocatalysis, pollution treatment, fine chemical synthesis, and phototheranostics.^[^ ^1^, ^2^, ^3^, ^4^, ^5^, ^6^, ^7^, ^8^, ^9^ ^]^ Although it is the most common approach to obtain elongated excitation wavelengths of photosensitizers by extending the conjugated systems, the difficulty of synthesis and the weak photo/chemical stability usually limit the general applications of the photosensitizers.^[^ ^10^, ^11^ ^]^ To circumvent such issues, the photon upconversion luminescent (UCL) technique has been proposed.^[^ ^12^, ^13^, ^14^ ^]^ In general, the characteristic of the UCL method is low energy photon absorption and high energy photon emission with a symbolic anti‐Stokes shift.^[^ ^15^, ^16^, ^17^, ^18^, ^19^ ^]^ With the largely elongated excitation wavelength, upconversion nanoparticles (UCNPs) and two‐photon absorption (TPA) materials have been extensively developed for cancer phototheranostics.^[^ ^20^, ^21^, ^22^, ^23^, ^24^, ^25^, ^26^ ^]^ However, the multi‐photon excitation needs one molecule simultaneous absorption of two or more photons to realize the anti‐Stokes process, which always requires extremely high excitation power density (e.g., >1 W cm^–2^) by a femtosecond laser light source.^[^ ^10^, ^27^, ^28^, ^29^, ^30^ ^]^ In addition, the unknown systematic toxicity and poor biocompatibility of inorganic materials further lessen the probability of clinical application.^[^ ^31^ ^]^

As a potential alternative, hot‐band absorption (HBA) UCL is a typical one‐photon process.^[^ ^32^, ^33^, ^34^ ^]^ Ordinarily, the phenomenon is characterized by the excitation of a molecule under vibrational–rotational energy levels and followed by the emission of radiation through Kasha's rule.^[^ ^32^, ^33^, ^35^ ^]^ Moreover, HBA UCL can be realized by simple dye molecules with high emission quantum yields, large molar extinction coefficients, and small Stokes shifts, of which the synthetic routes are simple.^[^ ^15^ ^]^ To develop HBA upconverted photosensitizers, a few researches have been proposed for photosensitization improvement by decorating Pd, Br, and I atoms to the HBA anti‐Stokes fluoresphores.^[^ ^11^, ^34^, ^36^, ^37^ ^]^ However, most of them only ameliorated the excited‐state intersystem crossing (ISC) by enhancing spin–orbit coupling (SOC) via heavy atom effect. HBA efficiency, another main contribution of HBA‐upconversion‐based photosensitization, has always been ignored, which largely limits upconverted photosensitization efficiency. Besides, the inherent drawbacks of heavy‐atom modification including enhanced dark cytotoxicity, weak stability, and high cost always plague researchers into transforming photosensitizers from laboratory to clinical use.^[^ ^38^, ^39^ ^]^

**Scheme 1:**
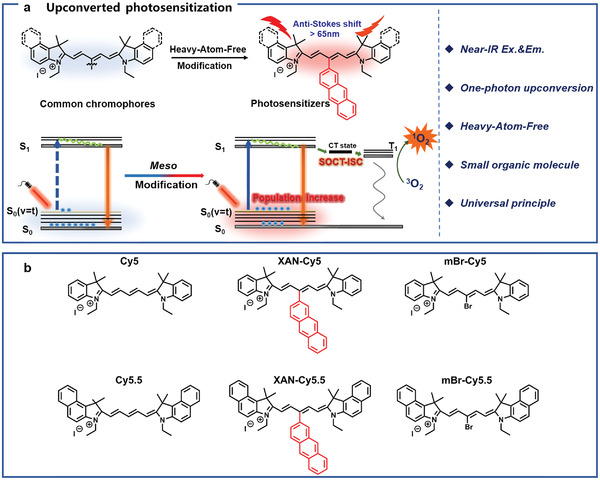
a) Development of single‐photon upconversion photosensitizers for deep tumor phototheranostic and b) Chemical structures of Cy5/Cy5.5‐based compounds.

Thus, we paid our attention to HBA efficiency improvement with heavy‐atom‐free structure. As one of the customized candidate of HBA UCL fluorophores, cyanine owns NIR excitation and emission wavelengths for deeper penetration depth.^[^ ^40^, ^41^, ^42^ ^]^ From the mechanismic point, the HBA efficiency can be enhanced by the richer populations under vibrational–rotational energy levels. Hence, XAN‐Cy5, a *meso* rotable anthracene modified pentamethine cyanine (Cy5) was tested under upconversion light (750 nm, 100 nm longer than its maximum absorption wavelength) excitation. To our surprise, XAN‐Cy5 exhibited an excellent 750 nm mediated ^1^O_2_ generation, which is even much higher than the heavy atom modified one (mBr‐Cy5). Temperature‐dependent absorption and emission spectra investigation and computational results indicated that the excellent upconversion excitation ability of XAN‐Cy5 results from the abundant populations in high vibrational–rotational energy levels. The efficient electron‐donating ability of anthracene could also cause the ISC improvement through the SOCT‐ISC mechanism, which further amplified ^1^O_2_ generation by 750 nm irradiation (**Scheme** **1**). Moreover, the Stokes shift and photostability of Cy5‐based chromophore are much improved under upconverted excitation. Another inspiring result is that a similar molecular modification could also be used for reference in designing Cy5.5‐based upconversion sensitizer (XAN‐Cy5.5), whose excitation wavelength can be elongated to 808 nm, and the photosensitization ability is comparable with heavy‐atom‐modified one. Benefited from the small cationic structure, the upconversion photosensitizer XAN‐Cy5 is capable of accumulating in mitochondria of cancer cells and causing their efficient destruction even under deep tissue. As for XAN‐Cy5.5, 808 nm light‐mediated in vivo UCL imaging exhibited improved signal‐to‐noise ratio (SNR) and anti‐disturbance of tissue heterogeneity compared with conventional fluorescence imaging. We believe that the systematic work can offer an instructive molecule design strategy for developing NIR upconversion light mediated ROS generation and luminescence.

## 2 Result and Discussion

### 2.1 Molecular Synthesis

To further elongate the excitation wavelength, Cy5.5 was chosen as the NIR HBA upconversion scaffold because it possesses an enlarged conjugated system and also a similar photophysical property to Cy5. We envisioned that the partially rotatable *2*‐anthracene at the *meso* position modification will also cause the upconversion excitation improvement. A series of cyanine derivatives were synthesized (Scheme 1b). Analogs of Cy5/Cy5.5 containing *meso* bromines were also synthesized as heavy‐atom controls. For XAN‐Cy5, XAN‐Cy5.5, anthracene‐based boronic acid moiety was introduced to the Cy5/Cy5.5 unit by Suzuki−Miyaura coupling reactions. For Cy5, mBr‐Cy5, Cy5.5, mBr‐Cy5.5, their indole units underwent Knoevenagel condensations. All the compounds were synthesized according to the synthetic routes detailed in supporting information. The chemical structures were fully confirmed by ^1^H NMR, ^13^C NMR, and ESI‐MS analytical data (Figures S37–S39, Supporting Information).

### 2.2 One‐Photon Upconversion Properties and Mechanism Explanation

**Figure 1:**
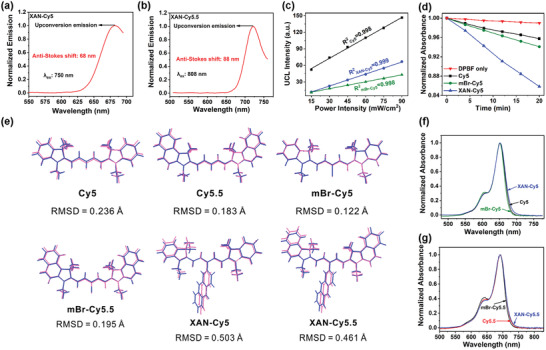
One‐photon upconversion photosensitization and verification for *meso* rotation. a) UCL spectrum of XAN‐Cy5 (2 µm) excited at 750 nm in dichloromethane (DCM) at 293 K. b) UCL spectrum of XAN‐Cy5.5 (2 µM) excited at 808 nm in DCM at 293 K. c) Upconversion light dose‐dependent UCL spectra of Cy5‐based compounds (2 µm) in DCM at 293 K. d) Normalized DPBF degradation (monitored at 415 nm) induced by different compounds (2 µm) under 750 nm light irradiation in DCM at 293 K. e) Overlaid ground state (pink) and excited state (blue) geometries of cyanine derivatives (optimized in vacuo) and the corresponding root‐mean‐square displacement of atoms. f) Normalized absorption spectra of Cy5‐based compounds (2 µm) in DCM at 293 K. g) Normalized absorption spectra of Cy5.5‐based compounds (2 µm) in DCM at 293 K.

The UCL spectra were found by exciting Cy5‐based compounds at 750 nm, which possess significant anti‐Stokes shifts of more than 65 nm (**Figure** **1**a and Figure S1, Supporting Information). More obviously, the intense NIR fluorescence emission with anti‐Stokes shifts about 90 nm was realized by exciting Cy5.5‐based compounds at 808 nm (Figure 1b and Figure S2, Supporting Information), indicating that the Cy5.5‐based compounds are more fit to be used as fluorescent sensors with improved signal‐to‐noise ratios (SNRs). The linear relationships between excitation light doses and emission intensities demonstrated that the UCL phenomena in Cy5 and Cy5.5 analogs are one‐photon processes (Figure 1c and Figure S2d, Supporting Information).

**Figure 2:**
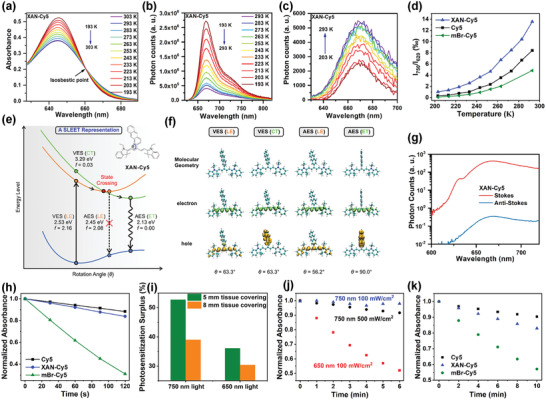
Photophysical test, verification of intramolecular SOCT‐ISC and the advantages of upconversion excitation. a) Temperature‐dependent UV–vis–NIR absorption spectra of XAN‐Cy5 (2 µm) in ethanol. b) Temperature‐dependent emission spectra of XAN‐Cy5 (2 µm) excited at 620 nm in ethanol. c) Temperature‐dependent emission spectra of XAN‐Cy5 (2 µm) excited at 750 nm in ethanol. d) Relative upconversion efficiency (*I*_750_/*I*_620_, the fluorescence intensity ratio between excitation light of 750 nm and that of 620 nm) of Cy5‐based compounds at different temperatures. e) The schematic illustration of the “state‐crossing from a locally excited to an electron‐transfer state” (SLEET) model and calculated excitation/de‐excitation energy (as well as oscillator strength *f*) of XAN‐Cy5 in DCM. f) Optimized molecular structures of XAN‐Cy5 in the ground and excited states, as well as the corresponding electron and hole distributions in DCM. VES and AES denote the vertically excited state and the adiabatic excited state, respectively. g) Stokes fluorescence emission and anti‐Stokes fluorescence emission spectra after excitation by the OPA light source in ethanol. h) Normalized DPBF degradation (monitored at 415 nm) induced by different compounds under 650 nm light irradiation in DCM at 293 K. i) Normalized DPBF degradation (monitored at 415 nm) induced by XAN‐Cy5 under different conditions in DCM at 293 K. j) Normalized XAN‐Cy5 degradation (monitored at 650 nm) under different conditions in water at 293 K. k) Normalized Cy5‐based compounds degradation (monitored at 650 nm) by different compounds under white light irradiation in water at 293 K.

Then the ^1^O_2_ generations of the compounds were tested under upconversion light irradiations (750 and 808 nm). Surprisingly, XAN‐Cy5 exhibited an enhanced ^1^O_2_ generation even compared with heavy atom modified mBr‐Cy5 (Figure 1d and Figures S3 and S4, Supporting Information). This result is completely contrary to the tests under excitation at the maximum absorption wavelength, during which, mBr‐Cy5 exhibited more than threefold higher ^1^O_2_ generation than XAN‐Cy5 (**Figure** **2**h and Figure S3, Supporting Information). As for XAN‐Cy5.5, 808 nm light irradiation could also cause a significant ^1^O_2_ generation that is also slightly higher than heavy atom modified one (mBr‐Cy5.5, Figure S5, Supporting Information). These interesting phenomena led us to probe mechanisms for enhanced upconversion photosensitization by “XAN” modification. As shown in Scheme 1a, the upconversion photosensitization contains two physicochemical processes, HBA of *S*_0_(*v* = *t*) − *S*_1_ and ISC of *S*_1_–*T*_1_. The following experiments systematically investigate the effects of “XAN” modification toward these two physicochemical processes.

For the HBA of *S*_0_(*v* = *t*)−*S*_1_ process, the populations in high vibration–rotation levels are increased by *meso* substituent. Firstly, we found that XAN‐Cy5 exhibited much‐decreased fluorescence intensity compared with Cy5 (Φ_f_ = 6.7% for XAN‐Cy5 vs Φ_f_ = 31% for Cy5), which is likely due to its local vibration–rotation of the *meso* substituent. Density functional theory (DFT) and time‐dependent DFT (TD‐DFT) calculations showed that the *meso*‐substitutes in XAN‐Cy5 and XAN‐Cy5.5 experienced a large degree of rotations upon photoexcitation (Figures S6 and S7, Supporting Information). Similar results were also obtained by calculating the root‐mean‐square displacements (RMSD) of the compounds which are used to quantify the amplitude of molecule geometric relaxation. The RMSD values of XAN‐Cy5 and XAN‐Cy5.5 are much larger (greater than twofold) than the other derivatives (Figure 1e). These results indicated that XAN‐Cy5 and XAN‐Cy5.5 experienced much larger geometrical changes as a result of the rotary *meso*‐substituents. Moreover, we calculated (1) the Huang–Rhys (HR) factors and (2) reorganization energy as a function of normal mode wavenumbers in vacuo (Figures S8– S13, Supporting Information). HR factor reflects the modification of vibrational quanta during the electronic transitions; large HR factors (especially in the low‐frequency vibrational modes) are associated with significant non‐radiative decays.^[^ ^43^ ^]^ Similarly, significant reorganization energy (especially in the low‐frequency vibration region) typically affords low fluorescence quantum yield. Our results show that the incorporation of a rotary group at the *meso*‐position in cyanine frame results in a significant contribution of the low‐frequency vibration modes (<200 cm^–1^) to the total reorganization energy (Figure S14 and Table S1, Supporting Information). In the low‐frequency vibration regions (<200 cm^–1^), the HR factors of XAN‐Cy5 and XAN‐Cy5.5 are particularly large (≈15), in comparison to the remaining compounds (<7).

Secondly, the vibrational modes in XAN‐Cy5 and XAN‐Cy5.5 are also applicable to the ground state. Compared with the original cyanine dyes that possess limited vibronic coupling with narrow UV–VIS–NIR spectra, XAN‐Cy5 and XAN‐Cy5.5 exhibited broad absorption spectra (Figure 1f,g). These broad spectra indicate that the introduction of the rotary *meso*‐substitutions could enrich the conformations of XAN‐Cy5/XAN‐Cy5.5 in the ground state (due to additional vibrational modes), thus generating a long‐wavelength absorption band.

To confirm the upconversion process is mediated by HBA, temperature‐dependent emission and UV–VIS–NIR absorption spectra were tested in Cy5‐based compounds. As expected, the absorption spectra become sharper, namely the narrower FWHM (the full width half maximum) in low temperatures (Figure 2a and Figure S15, Supporting Information), because the fluorophores tend to distribute in the lower vibration–rotation states suppressing HBA of *S*_0_(*v* = *t*) − *S*_1_ as temperature decreases. The conventional emission intensity and UCL intensity were obtained by 620 nm (Figure 2b) and 750 nm (Figure 2c) excitations, respectively, in the temperature range from 193 to 293 K. Stokes emission exhibited a negative correlation with temperature (Figure 2b and Figure S16, Supporting Information), because high temperature promotes non‐radiative relaxations due to molecular vibrations and rotations. Note: we choose the excitation at the isosbestic point to exclude the influence from the varying absorbance.

The correlation of UCL intensity with the temperature should result from two aspects. Firstly, the absorbance increase with the increasing temperature (Figure 2a and Figure S15, Supporting Information), which means more *S*_1_ molecules can be produced for stronger HBA. Secondly, the photoluminescence quantum yield (PLQY) is decreasing with the temperature (Figure 2b and Figure S16, Supporting Information). The first and second factors result in positive and negative correlations, respectively. The overall trend is the positive one (Figure 2c and Figure S17, Supporting Information), which demonstrates the upconversion process of XAN‐Cy5 was significantly HBA‐dependent. Furthermore, the HBA effect is powerful enough to overcome the PLQY decreasing effect at high temperatures. Then the relative upconversion efficiency was qualitatively defined by the intensity ratio between anti‐Stokes emission and Stokes emission. As shown in Figure 2d, at any temperature, the relative UCL efficiency of XAN‐Cy5 was much higher than other Cy5‐based compounds. Correspondingly, the isosbestic point in XAN‐Cy5 temperature‐dependent absorption spectra is also the most red‐shifted (Figure 2a and Figure S14, Supporting Information), indicating that the rotational *meso*‐substituent indeed causes the improved upconverted excitation efficiency. Moreover, the quantitative upconversion “absorption cross‐section” (the molar extinction coefficients at 750 nm) was obtained by using optical parametric amplification (OPA) as excitation laser for emission detection (Figure 2g and Figure S17, Supporting Information). The molar extinction coefficients of the Cy5, mBr‐Cy5, and XAN‐Cy5 at upconversion excitation region (750 nm) were calculated as 82.3, 56.7, and 121.3 m ^–1^ cm^–1^, respectively. This result systematically demonstrated that the populations of XAN‐Cy5 in high vibrational–rotational energy levels were most enriched, by which, the HBA efficacy was significantly improved.

For ISC of *S*_1_ − *T*_1_: The large Δ*E*_S‐T_ (the gap between singlet and triplet excited state energies) and small spin–orbit coupling (SOC) in Cy5 lead to a slow ISC rate, which also yields a low ^1^O_2_ quantum yield upon upconversion light (750 nm) excitation (Figure 1d). As reflected by the energy levels of frontier molecular orbitals, XAN‐Cy5 possessed a small energy gap between HOMO‐1 and HOMO (Δ*Ε* < 0.6 eV), in contrast to the large gaps (>1.5 eV) of Cy5 and mBr‐Cy5 (Figures S19–S21, Supporting Information). According to our reports, the small energy gap (Δ*Ε* ≈ 0.6 eV) gives rise to the photo‐induced electron transfer (PET) process, after which, charge reorganization induced ISC (SOCT‐ISC) would improve the ^1^O_2_ generation.^[^ ^44^ ^]^ Indeed, subsequent excited‐state calculations showed that there exists a stable electron transfer (ET) state in XAN‐Cy5 (Figure 2e). This low‐lying ET state reduces Δ*E*_S‐T_ and enhances SOC via SOCT (Figure S22, Supporting Information). The enhanced ISC efficiency (improving the *S*_1_ − *T*_1_ efficiency) coupled with the richer populations in high vibrational–rotational states (improving the *S*_0_–*S*_1_ efficiency) of XAN‐Cy5 resulted in the remarkable ^1^O_2_ generation enhancement excited by upconversion light (750 nm). Different from XAN‐Cy5, the heavy atom effect plays a crucial role in increasing the ISC rate and ^1^O_2_ quantum yield in mBr‐Cy5 under the maximum absorption wavelength excitation (Figure 2h). However, due to the poor vibrational band in the ground state with a lower upconversion efficiency, mBr‐Cy5 could only lead to a slightly higher ^1^O_2_ generation in comparison to Cy5 when excited at 750 nm, which is much lower than that of XAN‐Cy5 (Figure 1d). Thus, by the “two‐step” contributions in both ground state and excited state, XAN‐Cy5 realizes a significant ^1^O_2_ generation efficiency improvement under upconversion excitation.

Substantial rotations of the *meso*‐anthracene (Figures S6 and S7, Supporting Information) could also be found in XAN‐Cy5.5, suggesting that the HBA is also remarkable. The calculated Δ*Ε* value of XAN‐Cy5.5 is 0.639 eV, which is slightly higher than 0.6 eV (Figure S24, Supporting Information). Indeed, the weak SOCT‐ISC effect in XAN‐Cy5.5 can hardly improve the ^1^O_2_ generation under its maximum absorption wavelength excitation compared with Cy5.5 (Figure S26, Supporting Information). However, when excited by 808 nm light, XAN‐Cy5.5 showed an efficient ^1^O_2_ generation yield that is slightly higher than heavy‐atom modified Cy5.5 (mBr‐Cy5.5) (Figure S5, Supporting Information). The above results indicate that the rotation of the *meso*‐anthracene was an important factor to enhance the HBA upconversion photosensitization efficiency.

### 2.3 The Advantages of One‐Photon Upconversion

The much improved ^1^O_2_ generation of XAN‐Cy5 mediated by 750 nm light led us to investigate the advantages of upconversion excitation. Firstly, upconversion mediated ^1^O_2_ generation under deep tissue was simulated in solution test by covering tissues with different thicknesses (Figure S31, Supporting Information). As shown in Figure 2i, when covered by 5 mm tissue, 650 nm light stimulated ^1^O_2_ generation of XAN‐Cy5 exhibited a 63.9% decrease of DPBF degradation. However, only a 47.3% decrease was obtained by upconversion light excitation. A similar result could also be obtained by the 8 mm tissue model (Figure 2i, Figures S27 and S28, Supporting Information). Another advantage of such a one‐photon upconversion system is the elevated photostability. Owing to the large molar extinct coefficient, Cy5‐based dyes usually exhibit weak photostability under 650 nm irradiation. As shown in Figure 2j, under 100 mW cm^−2^ light dose of 650 nm light irradiation, the absorbance of XAN‐Cy5 showed much decrease. However, the absorbance of XAN‐Cy5 in the 750 nm light group was almost no change. Besides, even the light dose of 750 nm light was increased to 500 mW cm^−2^, only a slight decrease of absorbance was found. Thus, the upconversion excitation is conducive to the enhancement of the photostability, which can improve the photosensitizer utilization in photo‐treatment. As far as the Cy5‐based compounds are concerned, heavy‐atom‐effect in mBr‐Cy5 caused a serious loss of stability under visible light irradiation. In contrast, the excellent photostability of XAN‐Cy5 under the same condition overwhelmingly improved such issues (Figure 2k).

### 2.4 In Vitro and In Vivo Upconverison PDT and Imaging

**Figure 3:**
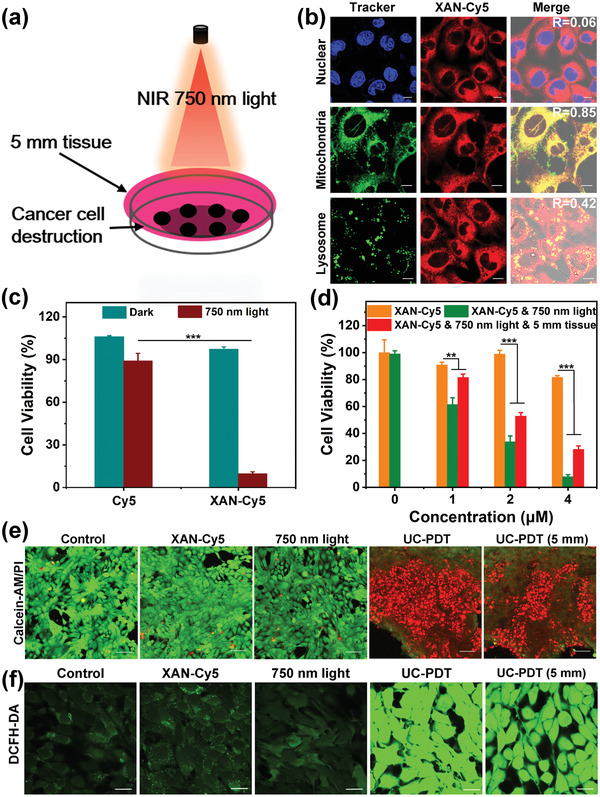
In vitro tests of XAN‐Cy5 under upconversion excitation mode. a) In vitro deep tissue phototherapeutic assay. b) Subcellular colocalization images of XAN‐Cy5 (1 µm) and commercial trackers. Scale bar = 10 µm. c) Cell viability of 4T1 cells treated with different compounds (2.5 µm) under dark or 750 nm light (500 mW cm^−2^, 15 min) irradiation. d) Cell viability of 4T1 cells treated with different dose of XAN‐Cy5 under dark or 750 nm light (500 mW cm^−2^, 15 min) irradiation. e) Calcein‐AM/PI stained 4T1 cells after different treatments. Scale bar = 50 µm. f) DCFH‐DA stained 4T1 cells after different treatments. Scale bar = 20 µm. Data were expressed as mean ± SD. **p < 0.01 and ***p < 0.001 determined by Student's *t*‐test.

Given the enhanced upconversion excitation efficiency and strong ^1^O_2_ generation potency of XAN‐Cy5, we conducted in vitro photoactivity experiments using murine mammary carcinoma 4T1 cells. Owing to the small cationic organic structure, XAN‐Cy5 could quickly accumulate in the mitochondria of 4T1 cells just within 1 h after incubation (Figure S29, Supporting Information and **Figure** **3**b). Under upconversion 750 nm light irradiation (500 mW cm^–2^, 15 min), XAN‐Cy5 could effectively cause the 4T1 cell destruction, with the half‐maximal inhibitory concentration (IC_50_) value as low as 1.33 µm (Figure 3d). Besides, the dark cytotoxicity is very low, more than 80% of cells were alive even if the concentration was up to 4 µm. Thanks to the efficient NIR light penetration and highly potential upconversion light‐mediated activation of XAN‐Cy5, we found that XAN‐Cy5 could also induce the 4T1 cell death even when the cell dish was covered by pork tissue of 5 mm thick, with the IC_50_ value of 2.16 µm (Figure 3a,d). In contrast, the anti‐cancer effects of Cy5 showed much decrease, which demonstrated that the upconversion PDT efficiency depends on ^1^O_2_ generation (Figure 3c). Furthermore, the intracellular behavior of XAN‐Cy5 under upconversion light irradiation was tested by using the confocal laser scanning microscopy (CLSM) mediated detection kit experiments. The intense intracellular green fluorescence of DCF in Figure 3f indicated that XAN‐Cy5 can effectively generate ^1^O_2_ whether the cell dish was covered by 5 mm tissue or not. The efficient photosensitization ability of XAN‐Cy5 resulted in the subsequent cell destruction since the intense intracellular red fluorescence of propidium iodide (dead cell) and weak green fluorescence of calcein‐AM (live cell) were obtained in photo‐treatment groups (Figure 3e).

**Figure 4:**
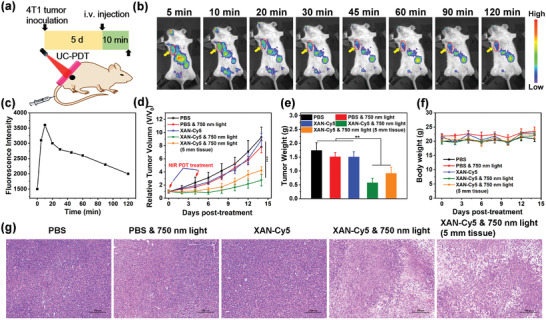
In vivo PDT tests of XAN‐Cy5 under upconversion excitation mode. a) In vivo deep tissue phototherapeutic assay. b) In vivo real‐time fluorescence imaging of 4T1 tumor‐bearing mice after i.v. injection of XAN‐Cy5 (100 µm). c) Relative fluorescence intensity of XAN‐Cy5 (100 µm) in 4T1 tumors at different time points. d) Relative tumor volume of mice after different treatments. e) Average tumor weight from each group after the whole treatment. f) Body weights of the mice after different treatments. g) H&E staining of tumor sections from different treatment groups after 14 days of treatment. Scale bar = 100 µm. Data were expressed as mean ± SD. **p < 0.01 and ***p < 0.001 determined by Student's *t*‐test.

The superior in vitro anti‐cancer effect encouraged us to examine the feasibility of XAN‐Cy5 for in vivo deep‐seated tumor PDT (**Figure** **4**a). Firstly, the in vivo fluorescence imaging was investigated at different time points after injection of XAN‐Cy5 intravenously when the tumor volume was reached at around 100 mm^3^. Because of the natively cationic frame and small molecular structure with good solubility, the tumor site is readily distinguishable from neighboring tissues after only 5 min of intravenous injection (Figure 4b). Simultaneously, XAN‐Cy5 started to accumulate and metabolize in the liver. As the fluorescence intensity of XAN‐Cy5 in the tumor site peaked at 10 min after injection (Figure 4c and Figure S30, Supporting Information), PDT treatment was carried out at this point.

As shown in Figure 4d, after twice of upconversion‐PDT treatment of XAN‐Cy5 (750 nm, 500 mW cm^−2^, 15 min), the group exhibited an extraordinary tumor regression. Surprisingly, the tumors were also effectively inhibited by XAN‐Cy5‐based upconversion‐PDT even when they were covered with a 5 mm tissue, demonstrating the excellent upconversion photosensitivity of XAN‐Cy5 for deep tumor therapy (Figure 4d and Figure S32, Supporting Information). Furthermore, the tumor weights, corresponding tumor photographs, and tumor hematoxylin and eosin (H&E) staining results validated the excellent antitumor results of XAN‐Cy5‐based upconversion‐PDT again (Figure 4e,g and Figure S33, Supporting Information). Thanks to the heavy‐atom‐free small organic molecule characteristic of XAN‐Cy5 with good biocompatibility and applicability, we did not observe any noticeable cell necrosis or inflammation lesions in any of the major organs, including the heart, liver, spleen, lungs, or kidneys (Figure S34, Supporting Information), and none of the mice displayed any abnormal body weight changes (Figure 4f).

**Figure 5:**
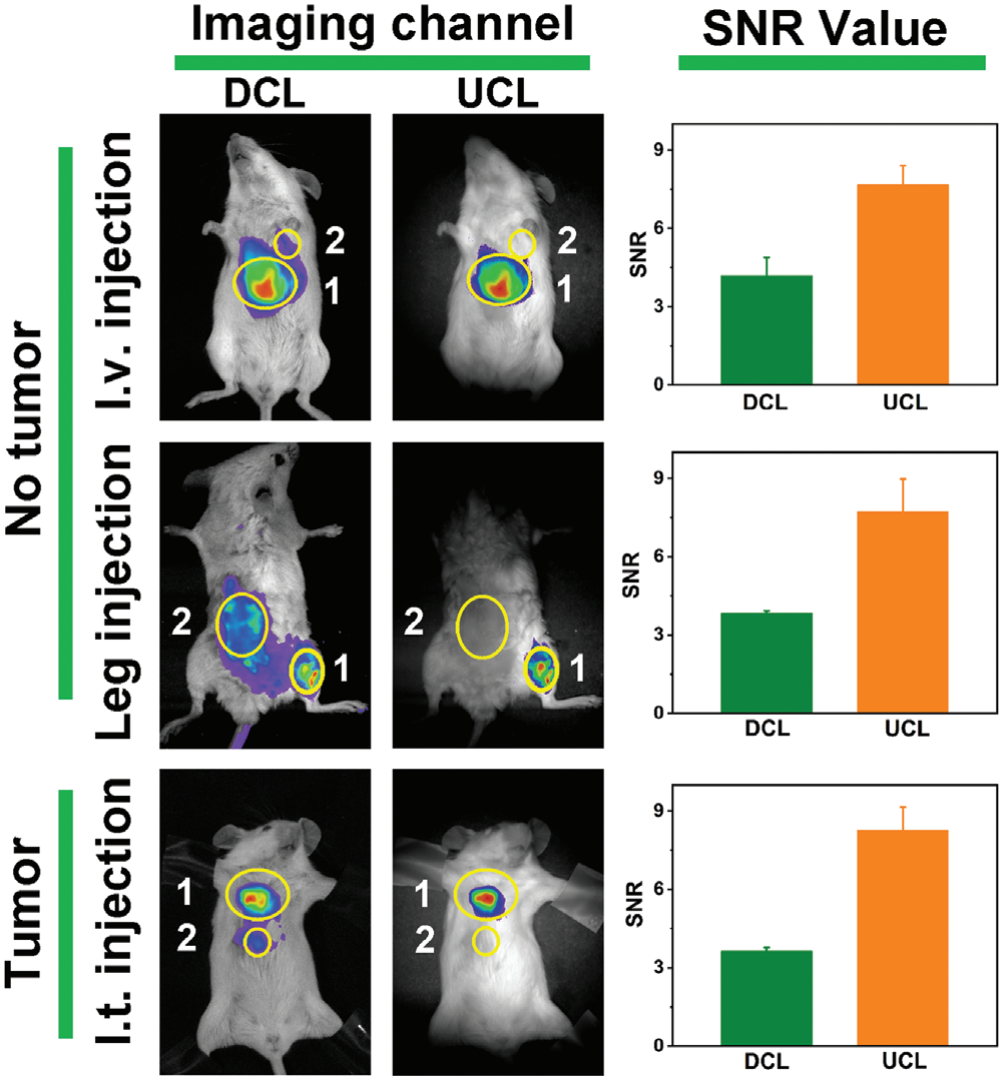
The comparison of down conversion luminescent (DCL) imaging and UCL imaging of XAN‐Cy5.5 (50 µm) in different in vivo models.

For XAN‐Cy5.5, the in vivo intravenous injection was firstly investigated in tumor‐bearing mice. As shown in Figures S35 and S36 (Supporting Information), the XAN‐Cy5 analog could also accumulate in tumor region at 120 min post‐injection. Then in vivo UCL imaging of XAN‐Cy5.5 was investigated by comparison with conventional fluorescence imaging (**Figure** **5**). As expected, owing to the larger Stokes shift of XAN‐Cy5.5 in UCL imaging than in conventional imaging (88 vs 28 nm), the targeted imaging region in the UCL group exhibited a nearly twofold increase of SNR no matter by intravenous injection of no‐tumor bearing mice or intratumoral injection of tumor‐bearing mice. Furthermore, as shown in Figure 5, when the mouse was shaved in the back region, the conventional fluorescence imaging offered the intense false positive signal in such area after the mouse was injected with XAN‐Cy5.5 in the right leg, causing the low in situ imaging SNR. In contrast, UCL imaging of XAN‐Cy5.5 obtained clear leg in situ fluorescence imaging. Thus, the NIR UCL imaging mode indeed overcame the issue of conventional imaging in which the imaging result was substantially altered by tissue heterogeneities and depth location. These biological application results indicated that the newly designed upconversion photosensitizers realize not only the efficient deep tissue PDT treatment but clear fluorescence imaging without the interference of tissue heterogeneities.

## 3 Conclusion

In summary, we found the excellent upconverted photosensitization of XAN‐Cy5, which exhibited much improved ^1^O_2_ generation even compared with the heavy atom modified frame under 750 nm light irradiation. By using temperature‐dependent spectroscopy study and computational chemistry, we rationalized the synergistic effect of improving vibration–rotation strength and electron donation by the substituent, which contributes to the enlarged HBA of *S*_0_(*v* = *t*) – S_1_ and ISC of *S*_1_–*T*_1_, respectively. Benefiting from such “two‐step” upconverted photosensitization improvement and heavy‐atom‐free structure, XAN‐Cy5 exhibits excellent deep tissue PDT potential and remains low cytotoxicity. The cationic cyanine backbone allows for improved hydrophilicity and biocompatibility, by which XAN‐Cy5 could accumulate within the tumor site and cause efficient deep tumor photoablation. To explore the usability of such modification, the similar molecular formation in Cy5.5‐based compound (XAN‐Cy5.5) was synthesized and tested. The excitation wavelength can be elongated to 808 nm, and the ROS generation ability is comparable with the heavy‐atom modified one. The intense 808 nm light meditated UCL imaging realized the improved SNR in in vivo imaging compared with conventional NIR fluorescence imaging. Our approach provides a new platform for heavy‐atom‐free one‐photon upconversion‐based cancer phototherapeutics. We also believe that our findings may meet the demands of other applications such as photocatalysis, synthetic chemistry, and pollution treatment.

## 4 Experimental Section

The general chemicals used in the report were purchased from Energy Chemical Co., Bide Pharmatech Ltd. and J&K Scientific Ltd., and all of the solvents were of analytic grade. DCFH‐DA (2,7‐dichlorofluorescein diacetate) Detection Kit and Calcein‐AM/PI Detection Kit were purchased from Beyotime Biotechnology Co. (China). All the other solvents and reagents used in this study were of analytical grade.

NMR spectra were detected by Bruker Avance II 400 and Bruker Avance III 500 spectrometers. Mass spectrometric (ESI‐MS) data were obtained with LTQ Orbit rap XL instruments. Absorption and emission spectra for all the compounds were performed with a Lambda 35 UV–visible spectrophotometer (PerkinElmer) and a VAEIAN CARY Eclipse fluorescence spectrophotometer (Serial No. FL0812‐M018), respectively. CLSM images were performed on Olympus FV3000 confocal laser scanning microscope. Small animals’ fluorescence imaging was carried out by NightOWL II LB983 living imaging system.

### Computational Methods

DFT and TD‐DFT were employed to rationalize the highly efficient single oxygen generation of designed cyanine dyes. Geometry optimizations in the ground and excited states were carried out with M06‐2X functional in combination with the Def2SVP basis set in vacuo and DCM, respectively. Frequency analysis was performed to confirm that stable structures on the potential energy surfaces were obtained. When the solvent effect (in DCM) was applicable, it was accounted for using the solvation model based on the density (SMD) model. In these calculations, the electronic energies of cyanine dyes in the excited state were calculated based on the corrected linear response (cLR) solvent formalism. All DFT/TD‐DFT calculations were carried out with Gaussian 16A.

The SOC between the singlet and triplet excited states was calculated with ORCA 4.1. Vibrational analysis was performed using MOMAP.

### Singlet Oxygen Detection

The singlet oxygen generated by the cyanine dyes was measured using 1,3‐diphenylisobenzofuran (DPBF). The absorbance of DPBF at 415 nm was adjusted to about 1.0 in dichloromethane. The cuvette was irradiated with 750 or 808 nm monochromatic light for various time, and absorption spectra were measured immediately.

### In Vitro Photo‐Cytotoxicity Assays

4T1 cells were seeded onto 96‐well plates at 5000 cells per well and incubated at 37 °C for 24 h. For normoxic photo‐cytotoxicity evaluation, different concentrations of Cy5‐based compound were added to the cell wells, respectively. Then, the cells were further incubated for 2 h. Subsequently, the cells were subjected to 750 nm light (500 mW cm^−2^, 15 min). Then the cells were further incubated for 12 h at 37 °C. Next, adding MTT solution (5 mg mL^−1^) in DMEM to each well. After incubating the cells for 4 h, the solution in each well was removed out carefully, and then adding 100 µL DMSO to each well, the absorbance at 490 nm was measured with a Bio‐Rad microplate reader. The cell viability was obtained by the following equation:

(1) $$\mathrm{Cell}\mathrm{viability}\left(\%\right)=\left(\frac{{\mathrm{OD}}_{\mathrm{PDT}}-{\mathrm{OD}}_{\mathrm{Black}\;\mathrm{control}}}{{\mathrm{OD}}_{\mathrm{Cont}\mathrm{rol}}-{\mathrm{OD}}_{\mathrm{Black}\;\mathrm{control}}}\right)\times 100\%$$

For dark toxicity measurement of Cy5 compounds, light irradiation step was canceled.

### Subcutaneous Tumor Model and In Vivo Imaging

The female BALB/c mice, 4–6 weeks of age, were purchased from Liaoning Changsheng Biotechnology Co., Ltd. This study was conducted in accordance with the Guide for the Care and Use of Laboratory Animals published by the US National Institutes of Health (8th edition, 2011). The animal protocol was approved by the local research ethics review board of the Animal Ethics Committee of Dalian University of Technology (Certificate number//Ethics approval no. is 2018‐043).

To establish subcutaneous tumor model, 5 × 10^6^ 4T1 cells were injected subcutaneously into the selected armpit positions to establish the solid tumor model of mice. Tumors were allowed to grow to about 100 mm^3^ in volume.

For in vivo tumor imaging, XAN‐Cy5 (10 nmol, 100 µL), XAN‐Cy5.5 (5 nmol, 100 µL) were intravenously injected into 4T1 tumor‐bearing BALB/c mice, respectively, and the fluorescence signals were monitored at different post‐injection time.

### Statistical Analysis

Data were expressed as mean ± standard deviation. Student's *t* test was used to evaluate the statistical significance. *P* values < 0.05 were regarded statistically significant (*p < 0.05, **p < 0.01, ***p < 0.001, ****p < 0.0001).

## Conflict of Interest

The authors declare no conflict of interest.

## Supporting information

Supporting Information

## Data Availability

The data that support the findings of this study are available from the corresponding author upon reasonable request.

## References

[1] T. Wang , S. Wang , Z. Liu , Z. He , P. Yu , M. Zhao , H. Zhang , L. Lu , Z. Wang , Z. Wang , W. Zhang , Y. Fan , C. Sun , D. Zhao , W. Liu , J.‐C. G. Bünzli , F. Zhang , Nat. Mater. 2021, 20, 1571. 34326504 10.1038/s41563-021-01063-7

[2] X. Zhao , S. Long , M. Li , J. Cao , Y. Li , L. Guo , W. Sun , J. Du , J. Fan , X. Peng , J. Am. Chem. Soc. 2020, 142, 1510. 31880443 10.1021/jacs.9b11800

[3] X. Zhao , J. Liu , J. Fan , H. Chao , X. Peng , Chem. Soc. Rev. 2021, 50, 4185. 33527104 10.1039/d0cs00173b

[4] C.‐L. Sun , J. Li , X.‐Z. Wang , R. Shen , S. Liu , J.‐Q. Jiang , T. Li , Q.‐W. Song , Q. Liao , H.‐B. Fu , J.‐N. Yao , H.‐L. Zhang , Chem 2019, 5, 600.

[5] M. H. Al‐Afyouni , T. N. Rohrabaugh , K. F. Al‐Afyouni , C. Turro , Chem. Sci. 2018, 9, 6711. 30310605 10.1039/c8sc02094a PMC6115629

[6] L. M. Loftus , K. F. Al‐Afyouni , C. Turro , Chem. ‐ Eur. J. 2018, 24, 11550. 29923260 10.1002/chem.201802405

[7] T. J. Whittemore , H. J. Sayre , C. Xue , T. A. White , J. C. Gallucci , C. Turro , J. Am. Chem. Soc. 2017, 139, 14724. 28976191 10.1021/jacs.7b08489

[8] Q. Ma , X. Sun , W. Wang , D. Yang , C. Yang , Q. Shen , J. Shao , Chin. Chem. Lett. 2022, 33, 1681.

[9] D. Chen , H. Dai , W. Wang , Y. Cai , X. Mou , J. Zou , J. Shao , Z. Mao , L. Zhong , X. Dong , Y. Zhao , Adv. Sci. 2022, 9, 2200128. 10.1002/advs.202200128 PMC9189669 35435332

[10] B. Zheng , D. Zhong , T. Xie , J. Zhou , W. Li , A. Ilyas , Y. Lu , M. Zhou , R. Deng , Chem 2021, 7, 1615.

[11] R. Tian , W. Sun , M. Li , S. Long , M. Li , J. Fan , L. Guo , X. Peng , Chem. Sci. 2019, 10, 10106. 32055365 10.1039/c9sc04034j PMC6991170

[12] S. Han , Z. Yi , J. Zhang , Q. Gu , L. Liang , X. Qin , J. Xu , Y. Wu , H. Xu , A. Rao , X. Liu , Nat. Commun. 2021, 12, 3704. 34140483 10.1038/s41467-021-23967-3 PMC8211736

[13] R. Duan , Y. Xu , X. Zeng , J. Xu , L. Liang , Z. Zhang , Z. Wang , X. Jiang , B. Xing , B. Liu , A. All , X. Li , L. P. Lee , X. Liu , Nano Lett. 2021, 21, 778. 33301328 10.1021/acs.nanolett.0c04520

[14] D. Mao , F. Hu , Z. Yi , Kenry , S. Xu , S. Yan , Z. Luo , W. Wu , Z. Wang , D. Kong , X. Liu , B. Liu , Sci. Adv. 2020, 6, 2712. 10.1126/sciadv.abb2712 PMC7319755 32637621

[15] X. Zhu , Q. Su , W. Feng , F. Li , Chem. Soc. Rev. 2017, 46, 1025. 27966684 10.1039/c6cs00415f

[16] N. Yanai , N. Kimizuka , Angew. Chem., Int. Ed. 2020, 59, 10252. 10.1002/anie.202001325 32092207

[17] F. Auzel , Chem. Rev. 2004, 104, 139. 14719973 10.1021/cr020357g

[18] X. Liu , R. Deng , Y. Zhang , Y. Wang , H. Chang , L. Huang , X. Liu , Chem. Soc. Rev. 2015, 44, 1479. 25693872 10.1039/c4cs00356j

[19] H. Xu , S. Han , R. Deng , Q. Su , Y. Wei , Y. Tang , X. Qin , X. Liu , Nat. Photonics 2021, 15, 732.

[20] W. Fan , P. Huang , X. Chen , Chem. Soc. Rev. 2016, 45, 6488. 27722560 10.1039/c6cs00616g

[21] N. M. Idris , M. K. Gnanasammandhan , J. Zhang , P. C. Ho , R. Mahendran , Y. Zhang , Nat. Med. 2012, 18, 1580. 22983397 10.1038/nm.2933

[22] D. B. L. Teh , A. Bansal , C. Chai , T. B. Toh , R. A. J. Tucker , G. G. L. Gammad , Y. Yeo , Z. Lei , X. Zheng , F. Yang , J. S. Ho , N. Bolem , B. C. Wu , M. K. Gnanasammandhan , L. Hooi , G. S. Dawe , C. Libedinsky , W.‐Y. Ong , B. Halliwell , E. K.‐H. Chow , K.‐L. Lim , Y. Zhang , B. K. Kennedy , Adv. Mater. 2020, 32, 2001459. 10.1002/adma.202001459 32484308

[23] F. Li , Y. Du , J. Liu , H. Sun , J. Wang , R. Li , D. Kim , T. Hyeon , D. Ling , Adv. Mater. 2018, 30, 1802808. 10.1002/adma.201802808 29999559

[24] L. Wu , J. Liu , P. Li , B. Tang , T. D. James , Chem. Soc. Rev. 2021, 50, 702. 33475109 10.1039/d0cs00861c

[25] A. V. Kachynski , A. Pliss , A. N. Kuzmin , T. Y. Ohulchanskyy , A. Baev , J. Qu , P. N. Prasad , Nat. Photonics 2014, 8, 455.

[26] H. A. Collins , M. Khurana , E. H. Moriyama , A. Mariampillai , E. Dahlstedt , M. Balaz , M. K. Kuimova , M. Drobizhev , V. X. D. Yang , D. Phillips , A. Rebane , B. C. Wilson , H. L. Anderson , Nat. Photonics 2008, 2, 420.

[27] D. Yang , P. A. Ma , Z. Hou , Z. Cheng , C. Li , J. Lin , Chem. Soc. Rev. 2015, 44, 1416. 24988288 10.1039/c4cs00155a

[28] S. Gai , C. Li , P. Yang , J. Lin , Chem. Rev. 2014, 114, 2343. 24344724 10.1021/cr4001594

[29] B. Zhou , B. Shi , D. Jin , X. Liu , Nat. Nanotechnol. 2015, 10, 924. 26530022 10.1038/nnano.2015.251

[30] Q. Su , S. Han , X. Xie , H. Zhu , H. Chen , C.‐K. Chen , R.‐S. Liu , X. Chen , F. Wang , X. Liu , J. Am. Chem. Soc. 2012, 134, 20849. 23210614 10.1021/ja3111048

[31] Y. Liu , Q. Su , M. Chen , Y. Dong , Y. Shi , W. Feng , Z.‐Y. Wu , F. Li , Adv. Mater. 2016, 28, 6625. 27185083 10.1002/adma.201601140

[32] S. Kumazaki , Chem. Phys. 2013, 419, 107.

[33] O. Dimitriev , A. Fedoryak , Y. Slominskii , A. Smirnova , T. Yoshida , Chem. Phys. Lett. 2020, 738, 136905.

[34] M. Drobizhev , A. Karotki , M. Kruk , A. Krivokapic , H. L. Anderson , A. Rebane , Chem. Phys. Lett. 2003, 370, 690.

[35] M. H. Bartl , B. J. Scott , G. Wirnsberger , A. Popitsch , G. D. C. P. C. Stucky , 2003, 4, 392. 10.1002/cphc.200390069 12728557

[36] X. Ju , L. Zhu , L. Li , C. Ye , Z. Liang , S. Chen , X. Wang , J. Mater. Chem. C 2021, 9, 6749.

[37] R. Tian , C. Wang , W. Chi , J. Fan , J. Du , S. Long , L. Guo , X. Liu , X. Peng , Chem. ‐ Eur. J. 2021, 27, 16707. 34648222 10.1002/chem.202102866

[38] X. Zhao , Q. Yao , S. Long , W. Chi , Y. Yang , D. Tan , X. Liu , H. Huang , W. Sun , J. Du , J. Fan , X. Peng , J. Am. Chem. Soc. 2021, 143, 12345. 34323480 10.1021/jacs.1c06275

[39] V.‐N. Nguyen , Y. Yan , J. Zhao , J. Yoon , Acc. Chem. Res. 2021, 54, 207. 33289536 10.1021/acs.accounts.0c00606

[40] W. Sun , S. Guo , C. Hu , J. Fan , X. Peng , Chem. Rev. 2016, 116, 7768. 27314280 10.1021/acs.chemrev.6b00001

[41] A. P. Gorka , R. R. Nani , M. J. Schnermann , Acc. Chem. Res. 2018, 51, 3226. 30418020 10.1021/acs.accounts.8b00384

[42] S. Zhu , Z. Hu , R. Tian , B. C. Yung , Q. Yang , S. Zhao , D. O. Kiesewetter , G. Niu , H. Sun , A. L. Antaris , X. Chen , Adv. Mater. 2018, 30, 1802546. 10.1002/adma.201802546 29985542

[43] G. Yu , S. Yin , Y. Liu , J. Chen , X. Xu , X. Sun , D. Ma , X. Zhan , Q. Peng , Z. Shuai , B. Tang , D. Zhu , W. Fang , Y. Luo , J. Am. Chem. Soc. 2005, 127, 6335. 15853340 10.1021/ja044628b

[44] W. Chi , J. Chen , W. Liu , C. Wang , Q. Qi , Q. Qiao , T. M. Tan , K. Xiong , X. Liu , K. Kang , Y.‐T. Chang , Z. Xu , X. Liu , J. Am. Chem. Soc. 2020, 142, 6777. 32182060 10.1021/jacs.0c01473

