# Supplementary material for: An Approach to Developing Cyanines with Upconverted Photosensitive Efficiency Enhancement for Highly Efficient NIR Tumor Phototheranostics
Source: Adv Sci (Weinh). 2022 Sep 12;9(31):2202885. doi: 10.1002/advs.202202885 (PMC9631065; doi:10.1002/advs.202202885)
Supplement: Supplementary file 1 — Supporting Information [file ADVS-9-2202885-s001.pdf]

ADVANCED SCIENCE
Open Access

Supporting Information

for *Adv. Sci.*, DOI 10.1002/advs.202202885

An Approach to Developing Cyanines with Upconverted Photosensitive Efficiency Enhancement for Highly Efficient NIR Tumor Phototheranostics

Xueze Zhao, Shan He, Weijie Chi, Xiaogang Liu, Pengzhong Chen, Wen Sun, Jianjun Du, Jiangli Fan and Xiaojun Peng*

Supporting Information

An Approach to Developing Cyanines with Upconverted Photosensitive Efficiency Enhancement for Highly Efficient NIR Tumor Phototheranostics

Xueze Zhao,[1] Shan He,[2] Weijie Chi,[3] Xiaogang Liu,[3] Pengzhong Chen,[1,4] Wen Sun,[1,4] Jianjun Du,[1,4] Jiangli Fan*,[1,4] and Xiaojun Peng[1,5]

[1] State Key Laboratory of Fine Chemicals, Frontiers Science Center for Smart Materials Oriented Chemical Engineering, Dalian University of Technology, Dalian 116024, China

[2] State Key Laboratory of Molecular Reaction Dynamics and Dynamics Research Center for Energy and Environmental Materials, Dalian Institute of Chemical Physics, Chinese Academy of Sciences, Dalian, Liaoning 116023, China

[3] Fluorescence Research Group, Singapore University of Technology and Design, 487372, Singapore

[4] Ningbo Institute of Dalian University of Technology, Ningbo 315016, China

[5] Shenzhen Research Institute, Dalian University of Technology, Shenzhen 518057, China

*e-mail: fanjl@dlut.edu.cn

EXPERIMENTAL PROCEDURES

Materials and Instrumentation

The general chemicals used in the report were purchased from Energy Chemical Co.,

Bide Pharmatech Ltd. and J&K Scientific Ltd., and all of the solvents were of analytic grade. DCFH-DA (2, 7-dichlorofluorescein diacetate) Detection Kit and Calcein-AM/PI Detection Kit were purchased from Beyotime Biotechnology Co. (China). All the other solvents and reagents used in this study were of analytical grade.

NMR spectra were detected by Bruker Avance II 400 and Bruker Avance III 500 spectrometers. Mass spectrometric (ESI-MS) data were obtained with LTQ Orbit rap XL instruments. Absorption and emission spectra for all the compounds were performed with a Lambda 35 UV-visible spectrophotometer (PerkinElmer) and a VAEIAN CARY Eclipse fluorescence spectrophotometer (Serial No. FL0812-M018), respectively. Confocal laser scanning microscope (CLSM) images were performed on Olympus FV3000 confocal laser scanning microscope. Small animals' fluorescence imaging was carried out by NightOWL II LB983 living imaging system.

Synthesis of cyanine dyes

The synthesis and structural characterization of the unreported cyanine dyes are shown in Scheme S1 and Figures S37-S39.

Computational Methods

Density functional theory (DFT) and time-dependent DFT (TD-DFT) were employed to rationalize the highly efficient single oxygen generation of designed cyanine dyes. Geometry optimizations in the ground and excited states were carried out with M06-2X functional[1] in combination with the Def2SVP basis set in vacuo and

dichloromethane, respectively. Frequency analysis was performed to confirm that we have obtained stable structures on the potential energy surfaces. When the solvent effect (in dichloromethane) was applicable, it was accounted for using the solvation model based on the density (SMD) model.[2] In these calculations, the electronic energies of cyanine dyes in the excited state were calculated based on the corrected linear response (cLR) solvent formalism. All DFT/TD-DFT calculations were carried out with *Gaussian 16A*.[3]

The spin-orbital coupling (SOC) between the singlet and triplet excited states was calculated with ORCA 4.1.[4, 5] Vibrational analysis was performed using *MOMAP*.[6]

Temperature dependent emission and absorption spectra

The temperature of the sample was controlled by a cryogenic temperature controller (Cryocon; model 22C). The controller has four-window ensure light transmittance. The absorption spectra were measured by a UV-VIS absorption spectrometer (Agilent Carry60). The emission spectra were observed by a static/transient fluorescence spectrometer (Edinburgh instruments; FLS 1000-stm). The excitation source is a Xe lamp with a monochromator. Along the axis perpendicular to the excitation light, the PL was collected by a lens, separated by a monochromator and finally detected by a PMT.

Determining the extinction coefficients in 750 nm and the Stokes and anti-Stokes fluorescence emission spectra

The fluorescence spectra apparatus is shown in scheme 1. The tunable excitation laser is the OPA pumped by a Ti:sapphire laser system (Coherent; 800 nm, 70 fs, 6 mJ/pulse, 1 kHz repetition rate). Along the axis perpendicular to the excitation laser, a lens system was used to collect the fluorescence and couple it into a fiber. The other side of the fiber was connected to a spectrometer (Maya2000 pro; Ocean Optics). Note: the fluorescence measure area is in the front of the cuvette to exclude the excitation power density disturbed by different absorbances of samples. A power meter (Thorlabs; S121C) was used to measure the power of excitation laser. We measured the emission spectra with the 620 nm and 750 nm laser excitation.

In order to determine the parameter A_{750}, the following deductions are made. Because of the Kasha's rule, the PLQY of a specific molecule is regardless of the excitation wavelength. Then we can write the Eqs. S1 and S2：

$$PLQY_{620} = k \times \frac{I_{620}}{\left(1-10^{-A_{620}}\right)\times P_{620}\times 620} \quad \text{(S1)}$$

$$PLQY_{750} = k \times \frac{I_{750}}{(1-10^{-A_{750}})\times P_{750}\times 750} \quad \text{(S2)}$$

Where the $I_{620,750}$ represent the fluorescence intensities with the 620 nm and 750 nm excitation, respectively. The $P_{620,750}$ are the laser power densities of 620 nm and 750 nm, respectively. The $A_{620,750}$ are the absorbances at 620 nm and 750 nm, respectively. Parameter k is the system correction factor. Consequently, the A_{750} can be calculated by eq. S3.

$$\frac{I_{620}}{(1-10^{-A_{620}})\times P_{620}\times 620} = \frac{I_{750}}{(1-10^{-A_{750}})\times P_{750}\times 750} \quad \text{(S3)}$$

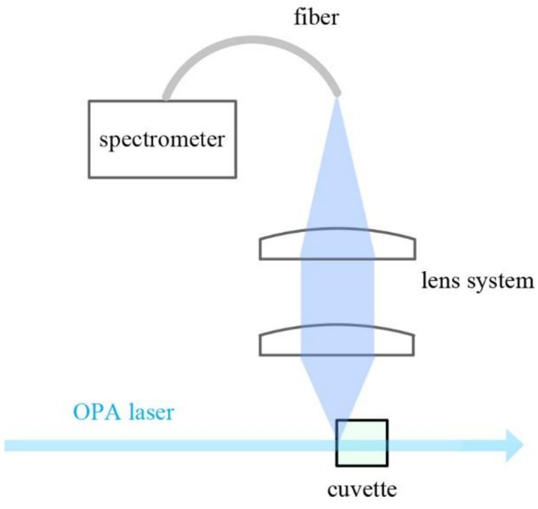

Scheme S1. Apparatus for fluorescence emission spectra

Singlet Oxygen Detection

The singlet oxygen generated by the cyanine dyes was measured using 1,3-diphenylisobenzofuran (DPBF). The absorbance of DPBF at 415 nm was adjusted to about 1.0 in dichloromethane. The cuvette was irradiated with 750 nm or 808 nm monochromatic light for various time, and absorption spectra were measured immediately.

Photostability Detection

The absorbance of Cy5 compounds at the 650 nm was monitored in water/DMSO (7:3). For **Cy5**, **XAN-Cy5** and **mBr-Cy5**, the cuvette was irradiated with white light for various time, and absorption spectra were measured immediately. For **XAN-Cy5** only, the cuvette was irradiated with 650 nm light and 750 nm for various time, respectively, and absorption spectra were measured immediately.

Cell and Culture Conditions

Mouse breast cancer cells 4T1 cells were maintained in DMEM medium, all of them were supplemented with 1% penicillinstreptomycin and 10% FBS, and atmosphere of 5% CO_2 and 95% air at 37 ºC. When used for imaging, all types of cells were cultured on 35 mm glass-bottom culture dishes for 12-24 h.

Confocal Fluorescence Imaging of Cells

The 4T1 cells were incubated for 24 h. Then **XAN-Cy5** (1 μM) was added, the confocal luminescence imaging was performed and images were collected. (excited at 640 nm, monitored at 660−710 nm). For cellular co-localization test, **XAN-Cy5** (1 μM) was added to the cells and incubated for 2 h, then the cells were further stained by LysoTracker Green DND 26 (100 nM), MitoTracker Green FM (100 nM), or Hoechst 33342 (100 nM). Cells were then visualized with laser confocal microscopy. The excitation wavelength for **XAN-Cy5** was 640 nm, while the excitation wavelength for LysoTracker Green DND 26 and MitoTracker Green FM were 488 nm, for Hoechst 33342 was 405 nm. The emission wavelength was collected from 660 to 710 nm for **XAN-Cy5**, 500 to 540 nm for Lyso Tracker Green and Mito Tracker Green, and 440 to 480 nm for Hoechst 33342.

In Vitro Photo-Cytotoxicity Assays

4T1 cells were seeded onto 96-well plates at 5000 cells per well and incubated at 37 ºC for 24 h. For normoxic photo-cytotoxicity evaluation, different concentrations of

Cy5-based compound were added to the cell wells, respectively. Then, the cells were further incubated for 2 h. Subsequently, the cells were subjected to 750 nm light (500 mW/cm^2, 15 min). Then the cells were further incubated for 12 h at 37ºC. Next, adding MTT solution (5 mg/mL) in DMEM to each well. After incubating the cells for 4 h, the solution in each well was removed out carefully, and then adding 100 μL DMSO to each well, the absorbance at 490 nm was measured with a Bio-Rad microplate reader. The cell viability was obtained by the following equation：

$$\text{Cell viability (\%)} = \left(\frac{\text{OD}_{PDT} - \text{OD}_{Black\ control}}{\text{OD}_{Control} - \text{OD}_{Black\ control}}\right) \times 100\%$$

For dark toxicity measurement of Cy5 compounds, light irradiation step was canceled.

Intracellular ROS Detection

DCFH-DA (2,7-dichlorofluorescein diacetate) Detection Kit was used to validate the generation of singlet oxygen in living 4T1 cells. The cells were firstly incubated on the cell culture plate for 24 h, then they were divided into five groups: 1) control cells; 2) cells under 10 min irradiation; 3) cells were incubated with 2.5 μM **XAN-Cy5** for 2 h; 4) cells were incubated with 2.5 μM **XAN-Cy5** for 2 h and irradiated with 750 nm light (500 mW cm^{-2}, 10 min); 5) cells were treated with 4) method and covered with a 5 mm tissue. DCFH-DA Detection Kit was used according to the manufacture instruction. Then, confocal luminescence imaging was performed (excited at 488 nm, monitored at 490−520 nm).

Confocal imaging of UC-PDT-induced cell death

4T1 cells were incubated on the cell culture plate for 24 h. Then they were exposed to different following treatments: 1) control cells; 2) cells under 10 min irradiation; 3) cells were incubated with 2.5 μM **XAN-Cy5** for 2 h; 4) cells were incubated with 2.5 μM **XAN-Cy5** for 2 h and irradiated with 750 nm light (500 mW cm^{-2}, 10 min); 5) cells were treated with 4) method and covered with a 5 mm tissue. After different treatments, cells were stained with Calcein AM/propidium iodide (PI) Apoptosis Detection Kit according to the manufacture instruction. The cell apoptosis was visualized by fluorescence microscopy with excitation wavelength of 488 nm. The emission wavelength was collected from 505 to 545 nm for green channel, and from 600 to 700 nm for red channel.

Subcutaneous tumor model and *in vivo* imaging

The female BALB/c mice, 4–6 weeks of age, were purchased from Liaoning Changsheng biotechnology co., Ltd. This study was conducted in accordance with the Guide for the Care and Use of Laboratory Animals published by the US National Institutes of Health (8th edition, 2011). The animal protocol was approved by the local research ethics review board of the Animal Ethics Committee of Dalian University of Technology (Certificate number//Ethics approval no. is 2018-043)

To establish subcutaneous tumor model, 5×10^6 4T1 cells were injected subcutaneously into the selected armpit positions to establish the solid tumor model of mice. Tumors were allowed to grow to about 100 mm^3 in volume.

For *in vivo* tumor imaging, **XAN-Cy5** (10 nmol, 100 μL), **XAN-Cy5.5** (5 nmol, 100

μL) were intravenously injected into 4T1 tumor-bearing BALB/c mice, respectively, and the fluorescence signals were monitored at different post-injection time.

Subcutaneous tumor *In vivo* PDT evaluation.

To confirm the *in vivo* PDT efficacy of **XAN-Cy5**, all mice were divided into five groups and subjected to treatments: group 1, PBS injection; group 2, PBS injection and irradiation; group 3, **XAN-Cy5** injection; group 4, **XAN-Cy5** injection and irradiation with 750 nm light; group 5, **XAN-Cy5** injection and irradiation with 750 nm light with 5 mm tissue covering. Each group contained five mice, and PBS (100 μL), **XAN-Cy5** (10 nmol, 100 μL) were injected intravenously. After 10 min post-injection, tumor region was irradiated with 750 nm light at a power density of 500 mW cm^{-2} for 15 min. In the following 2 weeks, the tumor volume of all mice was measured every two days using a vernier caliper. Then, the greatest longitudinal diameter (length) and the greatest transverse diameter (width) were used to calculate the tumor volume. Tumor volume = width × width × length/2. In addition, after 14 day post-first-treatment, tumor tissues of the abovementioned treatment groups 1-5 were harvested for histological analysis by means of hematoxylin-eosin (H&E) staining.

***In Vivo* Biosafety Assay**

The *in vivo* biosafety assay was performed by using measurement mice body weight and H&E slice histological analysis. At 14 days post-first-treatment, the mice were euthanized, and main organs including heart, liver, spleen, lung, kidneys were harvested for histological analysis by means of hematoxylin-eosin (H&E) staining.

Subcutaneous tumor *in vivo* upconversion imaging

For *in vivo* no-tumor imaging, **XAN-Cy5.5** (5 nmol, 100 μL) was intravenously injected into BALB/c mice without 4T1-tumor-bearing and the fluorescence signals were monitored at 30 min post-injection time under both two modes (excited by 808/655 nm lights).

For *in vivo* mice leg imaging, **XAN-Cy5.5** (5 nmol, 100 μL) was injected into BALB/c mice without 4T1-tumor-bearing and the fluorescence signals were monitored at 30 min post-injection time under both two modes (excited by 808/655 nm lights).

For *in vivo* tumor *in situ* imaging, **XAN-Cy5.5** (5 nmol, 100 μL) was intratumorally injected into BALB/c mice and the fluorescence signals were monitored at 30 min post-injection time under both two modes (excited by 808/655nm lights).

Statistical analysis

Data were expressed as mean ± standard deviation. Student's t test was used to evaluate the statistical significance. P values < 0.05 were regarded statistically significant (*p < 0.05, **p < 0.01, ***p < 0.001, ****p < 0.0001).

According to the literature methods. **Cy5**, **mBr−Cy5**, **XAN-Cy5**, **Cy5.5**, **mBr-Cy5.5** were prepared.[7-9]

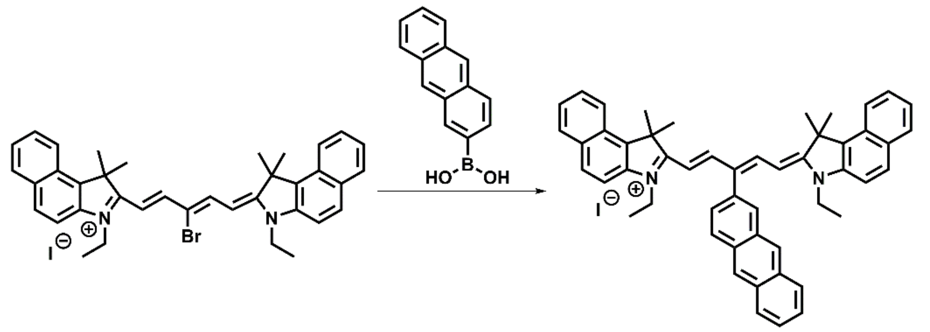

Scheme S2. Synthesis routes of **XAN-Cy5.5**. Reagents and conditions: $Pd(PPh_3)_4$, K_2CO_3, 1,4-dioxane, 90 °C, 6 h.

Synthesis of **XAN-Cy5.5**

mBr-Cy5.5 (0.1 g, 0.16 mmol), anthracene boric acid (0.056 g, 0.25 mmol), $Pd(PPh_3)_4$ (0.0092 g, 0.008 mmol), K_2CO_3 (0.066 g, 0.48 mmol) was dissolved in 10 mL 1,4-dioxane and 500 μL H_2O and stirred at 90°C for 6 h. Finally the solvents were evaporated and the mixture was purified by column chromatography with CH_2Cl_2/CH_3OH (100:0.8) as eluent (Yield = 15 %). ^1H NMR (600 MHz, DMSO-d6) δ 8.74 (s, 1H), 8.73 – 8.68 (m, 3H), 8.32 (d, J = 8.7 Hz, 1H), 8.29 (d, J = 8.5 Hz, 2H), 8.19 – 8.12 (m, 3H), 8.06 (d, J = 9.0 Hz, 4H), 7.71 (t, J = 7.7 Hz, 2H), 7.67 (d, J = 8.9 Hz, 2H), 7.61 – 7.57 (m, 2H), 7.55 – 7.49 (m, 3H), 5.90 (d, J = 14.2 Hz, 2H), 3.85 (q, J = 7.1 Hz, 4H), 2.07 (s, 12H), 1.08 (t, J = 7.3 Hz, 6H). ^{13}C NMR (126 MHz, CD_2Cl_2) δ(ppm): 174.55, 152.43, 139.32, 136.30, 134.73, 134.63, 132.74, 132.59, 132.56, 132.44, 132.19, 131.53, 131.04, 130.39, 130.03, 129.77, 128.76, 128.60, 128.54, 128.27, 128.08, 126.83, 126.75, 126.39, 126.36, 125.66, 122.77, 110.77, 101.19, 51.80, 30.11, 27.87, 12.50. ESI-MS ($C_{51}H_{47}IN_2$) m/z: $[M – I]^-$ calcd 687.3734, found 687.3722.

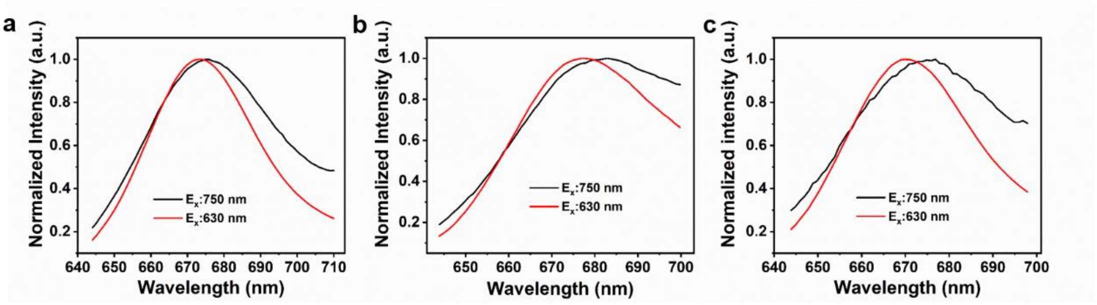

Figure S1. Emission spectra of Cy5-based compounds in dichloromethane. (a) **Cy5**. (b) **XAN-Cy5**. (c) **mBr-Cy5**.

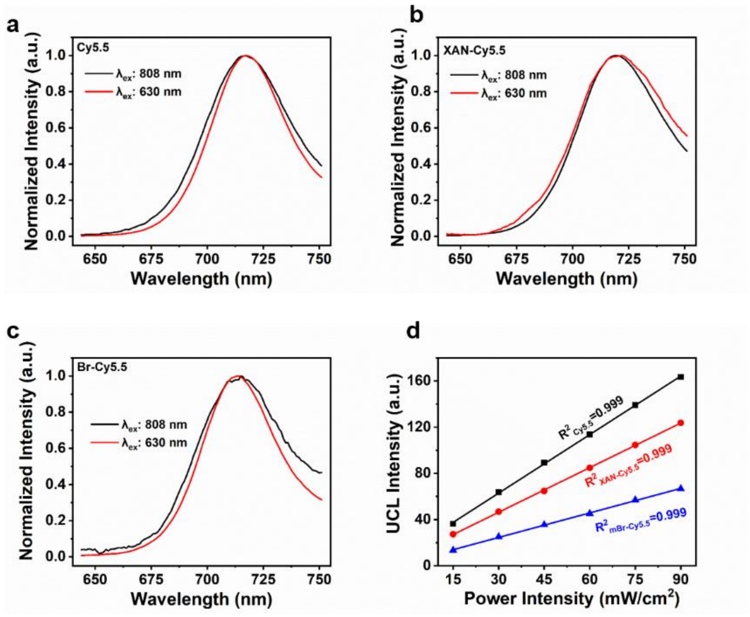

Figure S2. Upconversion emission and conventional emission of Cy5.5-based compounds excited by 808/630 nm light. (a) **Cy5.5**. (b) **XAN-Cy5.5**. (c) **mBr-Cy5.5**. (d) Upconversion light dose dependent UCL spectra of Cy5.5-based compounds.

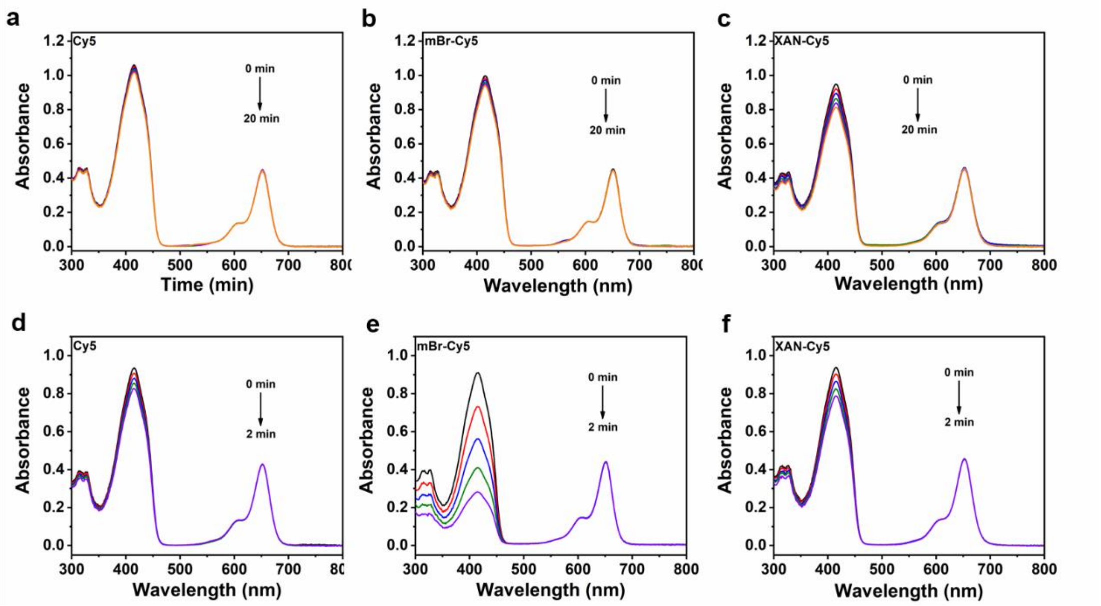

Figure S3. DPBF degradation (monitored at 415 nm) induced by different compounds under 750 nm (a, b, c) and 650 nm (d, e, f) light irradiations in dichloromethane.

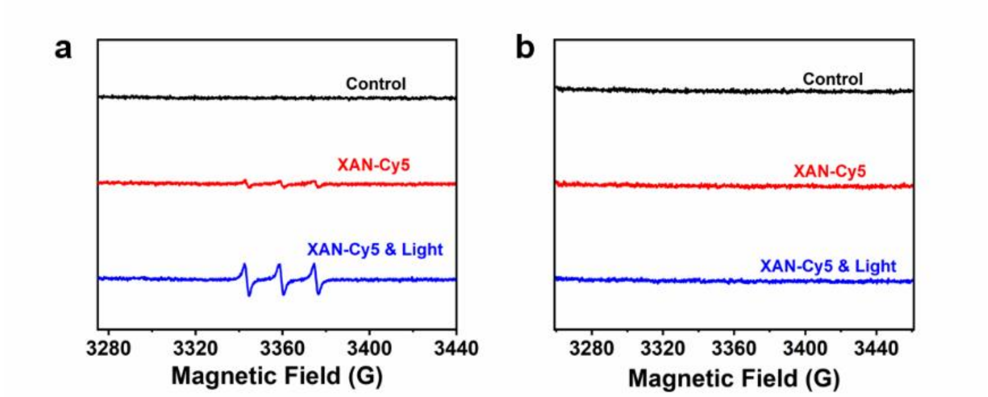

Figure S4. ESR tests of **XAN-Cy5** with TEMP (a) and DMPO (b) radical scavenger under upconversion light irradiation (500 mW/cm^2, 30 min) in dichloromethane for ROS type determination.

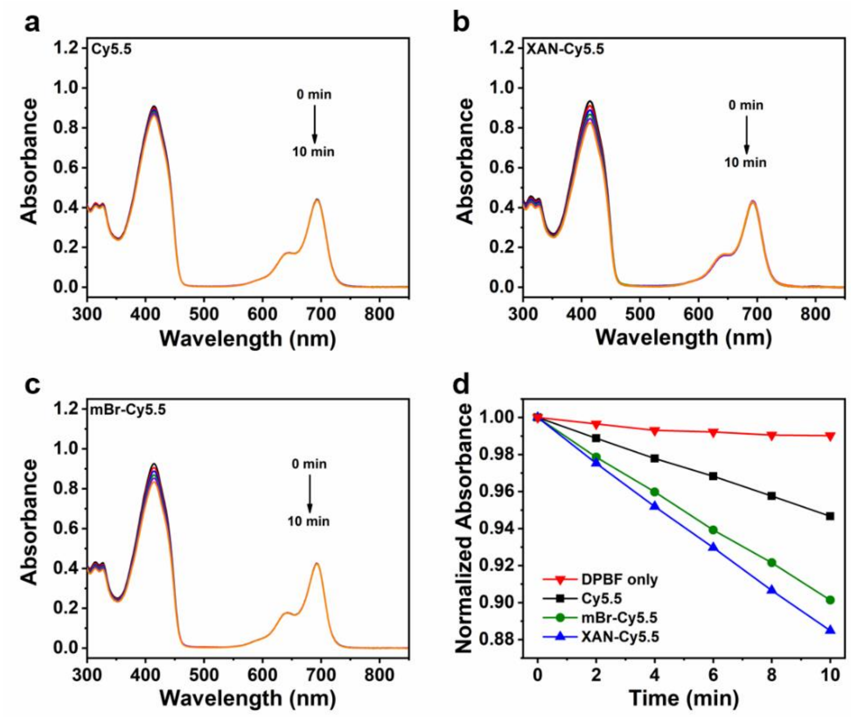

Figure S5. DPBF degradation (monitored at 415 nm) induced by different compounds under 808 nm light irradiation in dichloromethane. (a) **Cy5.5**, (b) **XAN-Cy5.5**, (c) **mBr-Cy5.5** (d) Normalized absorbance of DPBF at 415 nm after different compounds treatment.

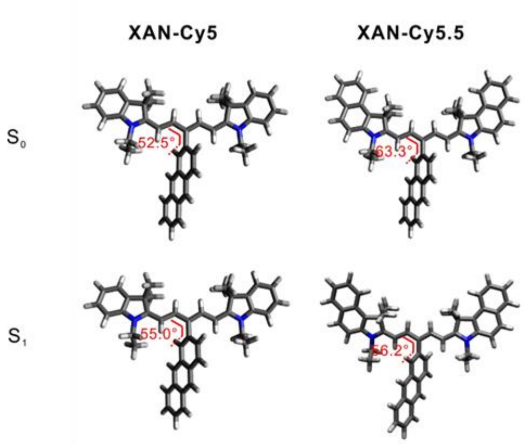

Figure S6. Optimized geometries of **XAN-Cy5**, and **XAN-Cy5.5** in the ground (S_0) and the first excited singlet state (S_1) in dichloromethane. The dihedral angle of the *meso*-substituent, θ, was highlighted in red, which demonstrated a considerable rotation upon photoexcitation.

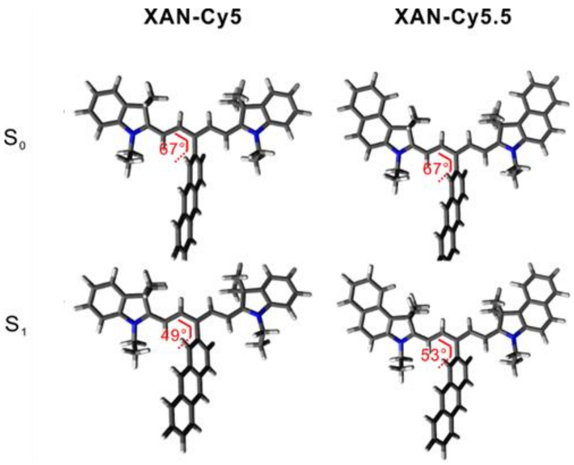

Figure S7. Optimized geometries of **XAN-Cy5**, and **XAN-Cy5.5** in the ground (S_0) and the first excited singlet state (S_1) in in vacuum. The dihedral angle of the *meso*-substituent, θ, was highlighted in red, which demonstrated a considerable rotation upon photoexcitation.

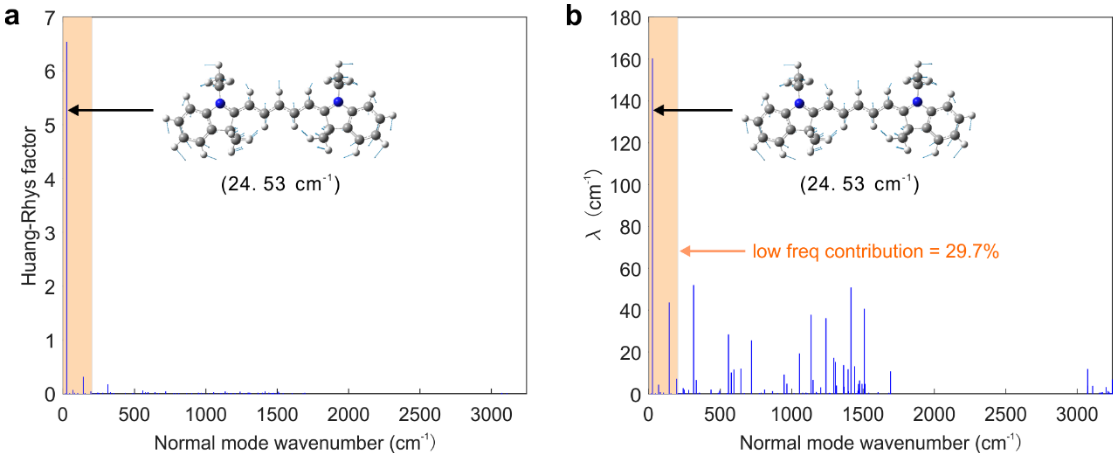

Figure S8. (a) Calculated Huang-Rhys factor and (b) reorganization energy of **Cy5** in the S_1 state in vacuo. The inset shows the major low-frequency vibration mode and corresponding frequency, as well as the low frequency (<200 cm^{-1}) contribution to the total reorganization energy.

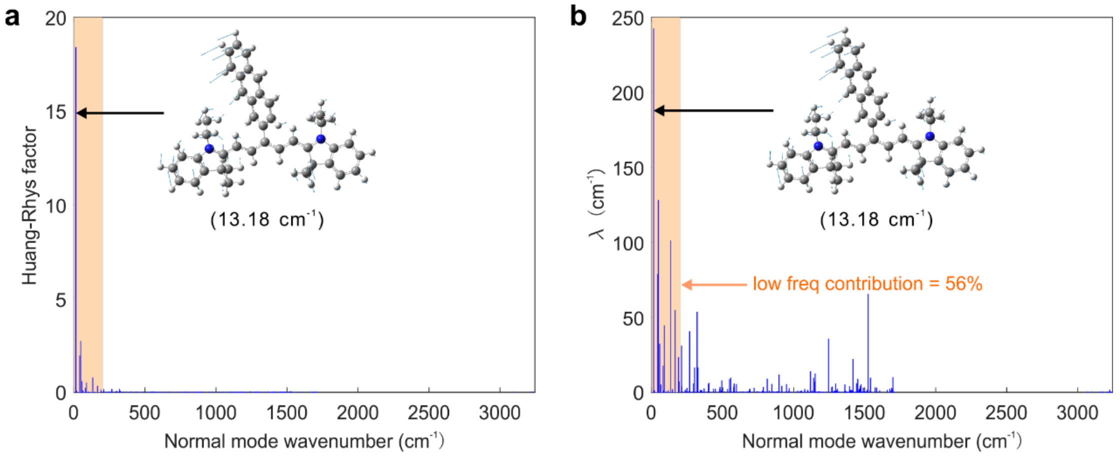

Figure S9. (a) Calculated Huang-Rhys factor and (b) reorganization energy of **XAN-Cy5** in the S_1 state in vacuo. The inset shows the major low-frequency vibration mode and corresponding frequency, as well as the low frequency (<200 cm^{-1}) contribution to the total reorganization energy.

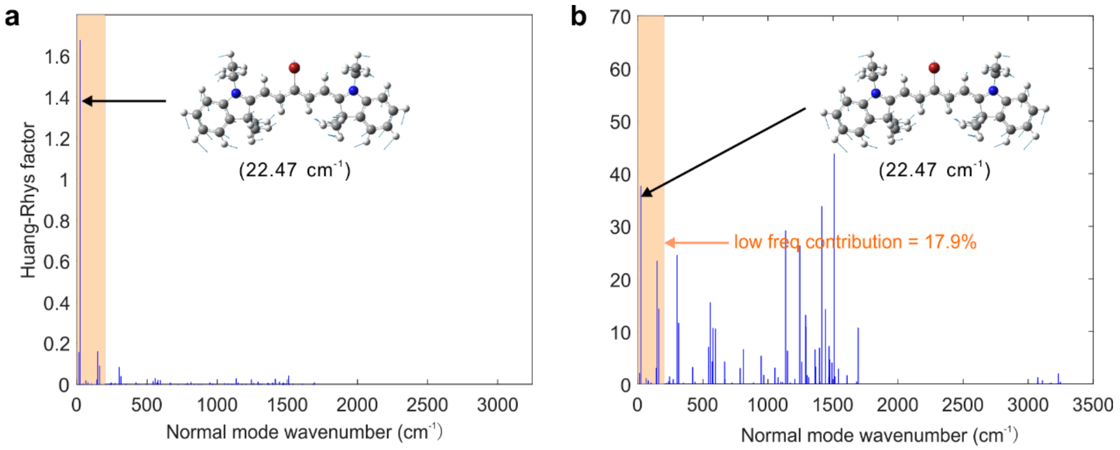

Figure S10. (a) Calculated Huang-Rhys factor and (b) reorganization energy of **mBr-Cy5** in the S_1 state in vacuo. The inset shows the major low-frequency vibration mode and corresponding frequency, as well as the low frequency (<200 cm^{-1}) contribution to the total reorganization energy.

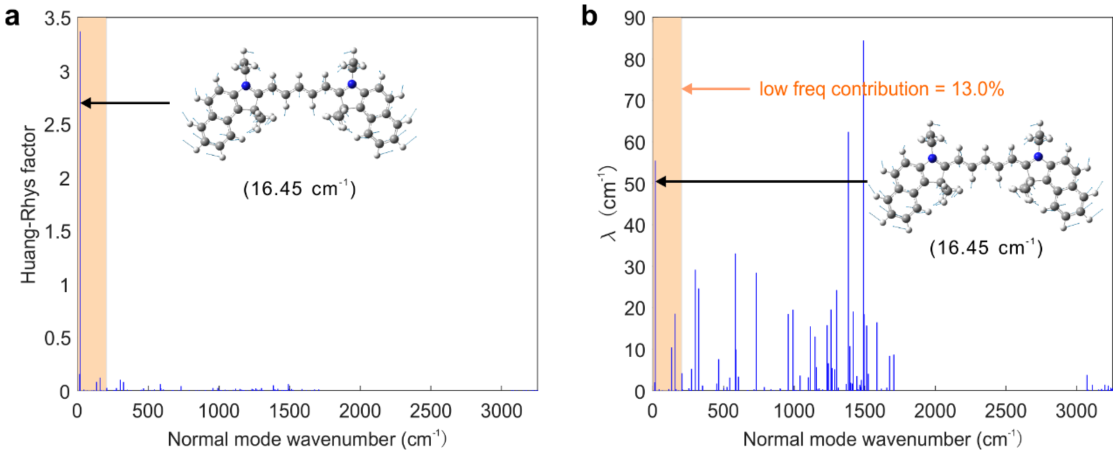

Figure S11. (a) Calculated Huang-Rhys factor and (b) reorganization energy of **Cy5.5** in the S_1 state in vacuo. The inset shows the major low-frequency vibration mode and corresponding frequency, as well as the low frequency (<200 cm^{-1}) contribution to the total reorganization energy.

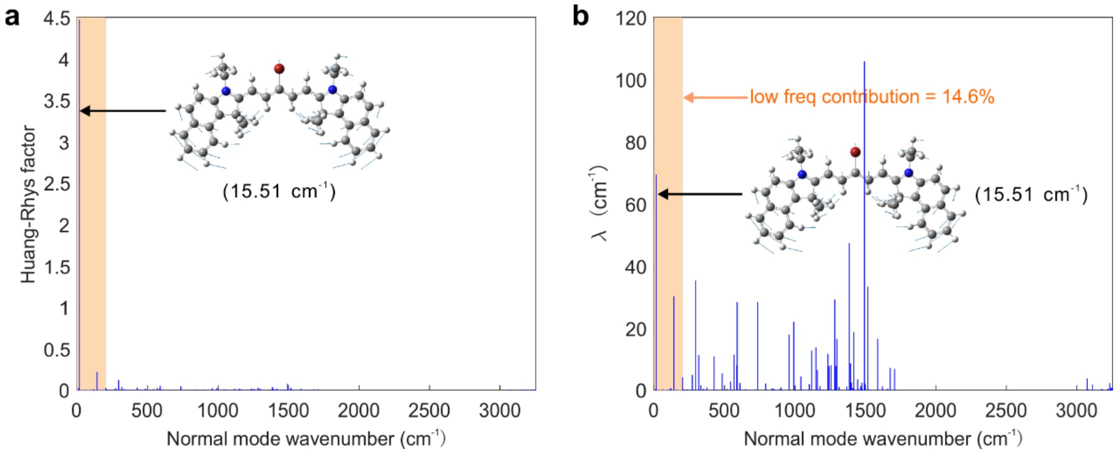

Figure S12. (a) Calculated Huang-Rhys factor and (b) reorganization energy of **mBr-Cy5.5** in the S_1 state in vacuo. The inset shows the major low-frequency vibration mode and corresponding frequency, as well as the low frequency (<200 cm^{-1}) contribution to the total reorganization energy.

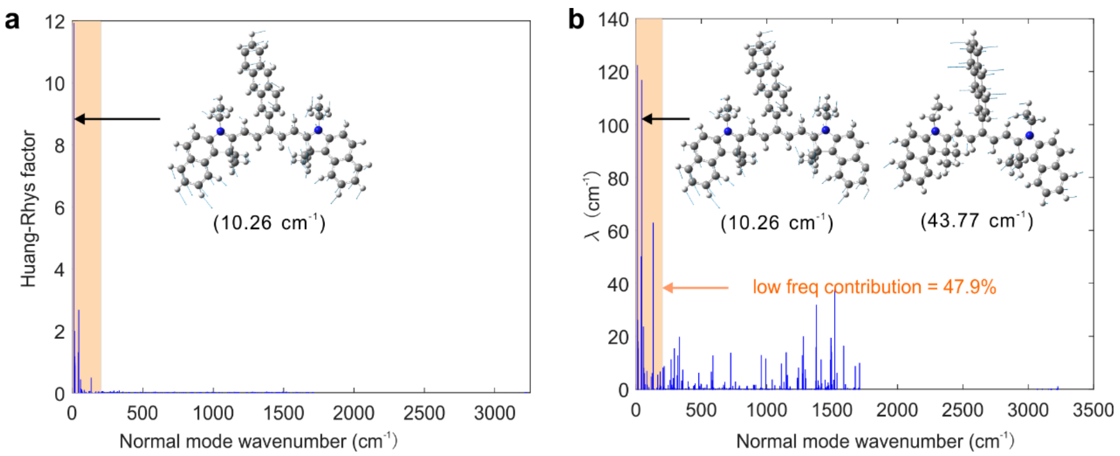

Figure S13. (a) Calculated Huang-Rhys factor and (b) reorganization energy of **XAN-Cy5.5** in the S_1 state in vacuo. The inset shows the major low-frequency vibration mode and corresponding frequency, as well as the low frequency (<200 cm^{-1}) contribution to the total reorganization energy.

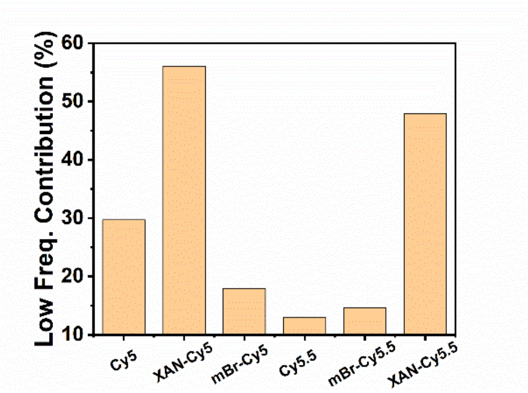

Figure S14. Comparison of contributions from the low-frequency vibration modes (<200 cm^{-1}) to the total reorganization energy in the excited state of cyanine derivatives in vacuo.

Table S1. Contributions of the low-frequency vibration modes (<200 cm^{-1}) on the total reorganization energy of cyanine derivatives in vacuo.

Compound	Cy5	XAN-Cy5	mBr-Cy5	Cy5.5	XAN-Cy5.5	mBr-Cy5.5
S_1 Low Freq. Contribution	29.7%	56.0%	17.9%	13.0%	47.9%	14.6%
S_0 Low Freq. Contribution	20.1%	38.8%	16.7%	12.0%	32.2%	12.0%

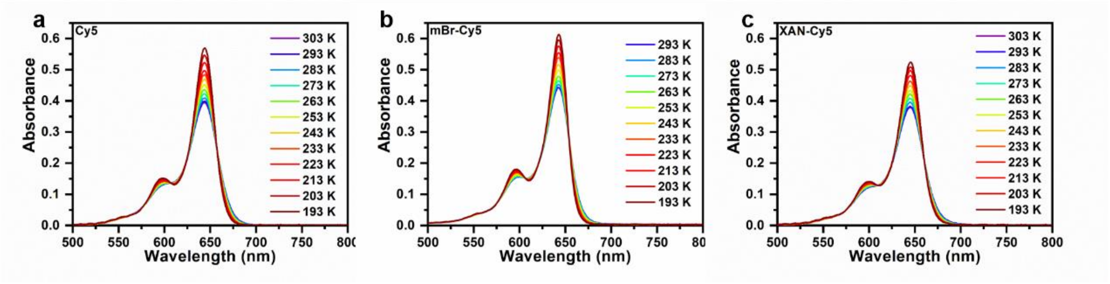

Figure S15. Temperature dependent absorption spectra of (a) Cy5, (b) mBr-Cy5 and (c) XAN-Cy5.

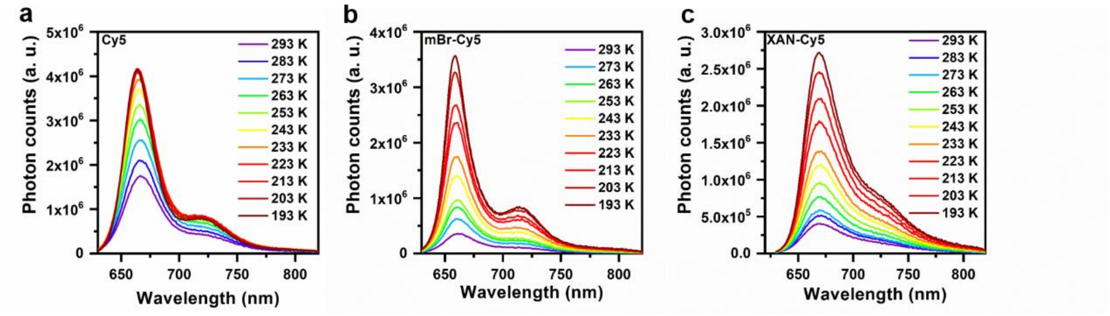

Figure S16. Temperature dependent fluorescence spectra of (a) Cy5, (b) mBr-Cy5 and (c) XAN-Cy5.

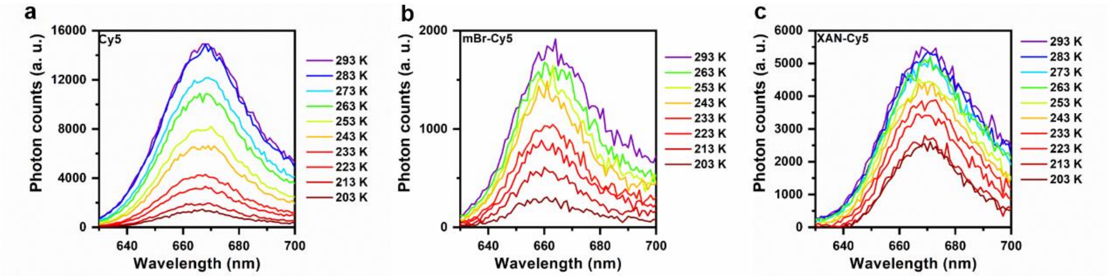

Figure S17. Temperature dependent upconversion fluorescence spectra of (a) Cy5, (b) mBr-Cy5 and (c) XAN-Cy5.

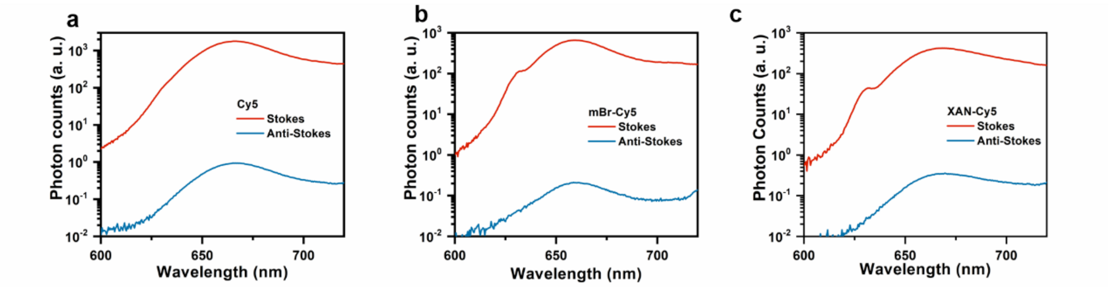

Figure S18. Upconversion fluorescence spectra and conventional fluorescence spectra of (a) Cy5, (b) mBr-Cy5 and (c) XAN-Cy5 excited by OPA light source.

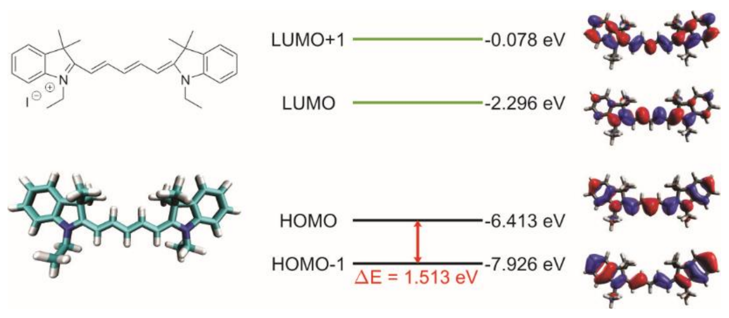

Figure S19. Molecular structures, optimized geometries, frontier molecular orbitals, corresponding energy levels, and calculated Δ*E* of **Cy5** in dichloromethane.

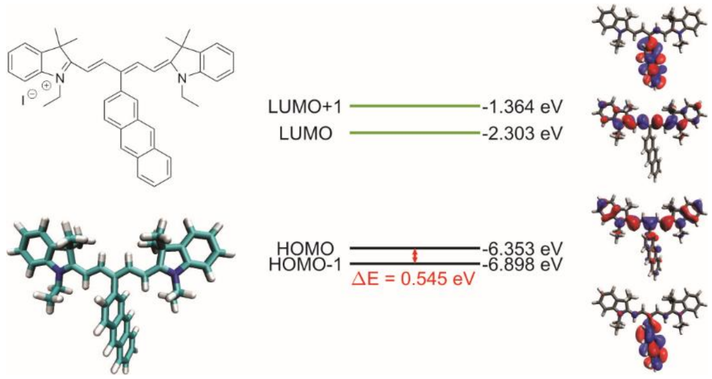

Figure S20. Molecular structures, optimized geometries, frontier molecular orbitals, corresponding energy levels, and calculated ΔE of **XAN-Cy5** in dichloromethane.

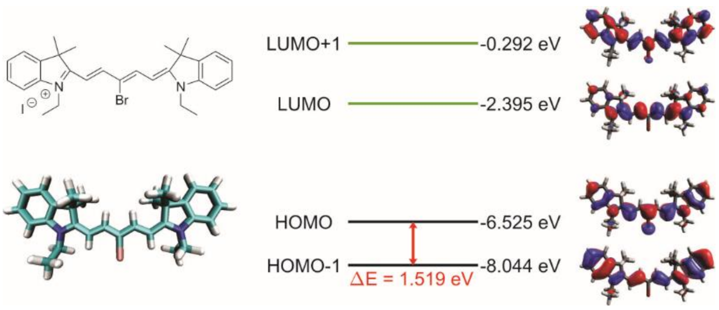

Figure S21. Molecular structures, optimized geometries, frontier molecular orbitals, corresponding energy levels, and calculated ΔE of **mBr-Cy5** in dichloromethane.

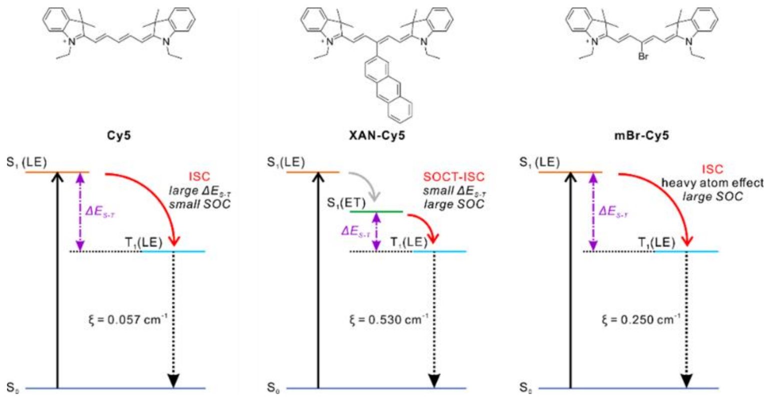

Figure S22. Calculated spin−orbital coupling values based on the geometries of the lowest-lying excited state (LE for **Cy5**, and **Br-Cy5**; and electron-transfer state for **XAN-Cy5**) in dichloromethane.

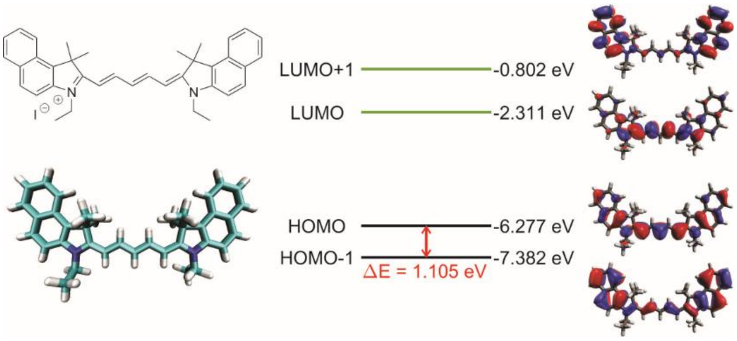

Figure S23. Molecular structures, optimized geometries, frontier molecular orbitals, corresponding energy levels, and calculated Δ*E* of **Cy5.5** in dichloromethane.

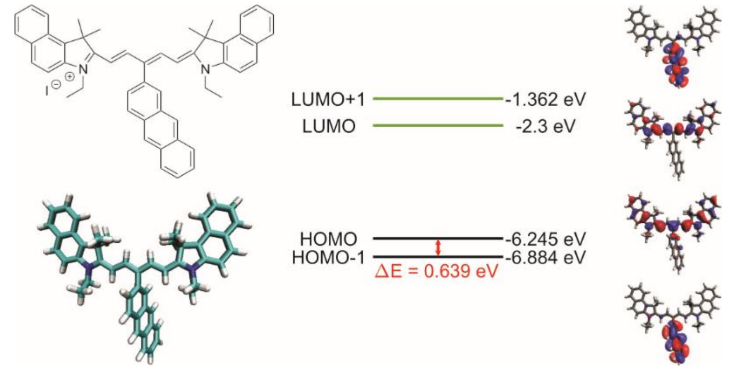

Figure S24. Molecular structures, optimized geometries, frontier molecular orbitals, corresponding energy levels, and calculated Δ*E* of **XAN-Cy5.5** in dichloromethane.

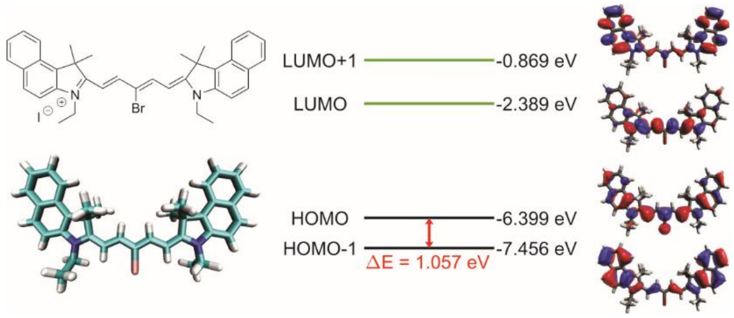

Figure S25. Molecular structures, optimized geometries, frontier molecular orbitals, corresponding energy levels, and calculated Δ*E* of **mBr-Cy5.5** in dichloromethane.

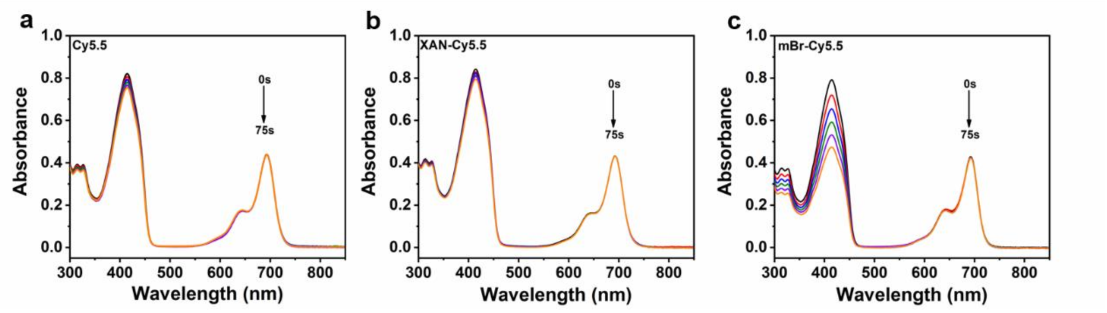

Figure S26. DPBF degradation (monitored at 415 nm) induced by different compounds under 700

nm light irradiation in dichloromethane. (a) **Cy5.5**, (b) **XAN-Cy5.5**, (c) **mBr-Cy5.5**.

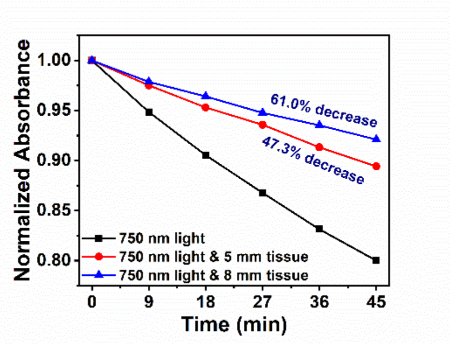

Figure S27. DPBF degradation (monitored at 415 nm) induced by XAN-Cy5 under 750 nm light irradiation in dichloromethane.

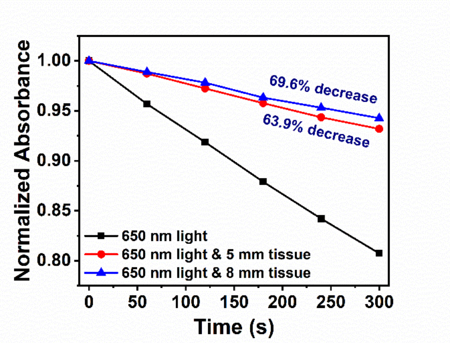

Figure S28. DPBF degradation (monitored at 415 nm) induced by XAN-Cy5 under 650 nm light irradiation in dichloromethane.

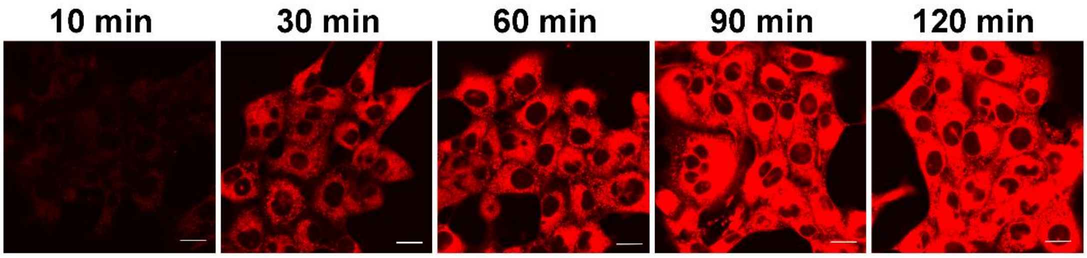

Figure S29. Cellular uptake of **XAN-Cy5** in 4T1 cells (λ_{ex}: 640 nm, λ_{em}: 660–710 nm, scale bars: 20 μm).

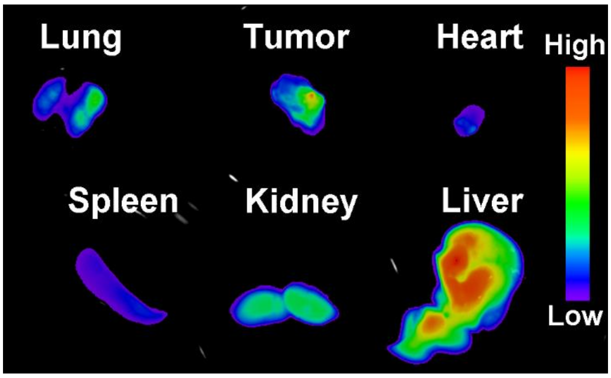

Figure 30. *Ex vivo* imaging of major organs, including heart, liver, spleen, lung, kidney and tumor at 10 min post *i.v.* injection of **XAN-Cy5**.

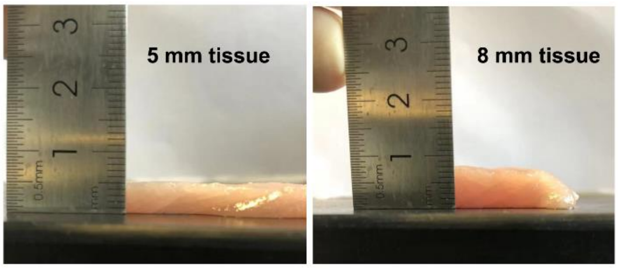

Figure S31. Typical images of experimental pork tissues.

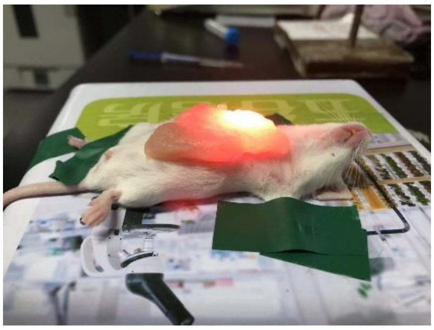

Figure S32. Typical images of *in vivo* deep seated tumor upconversion PDT.

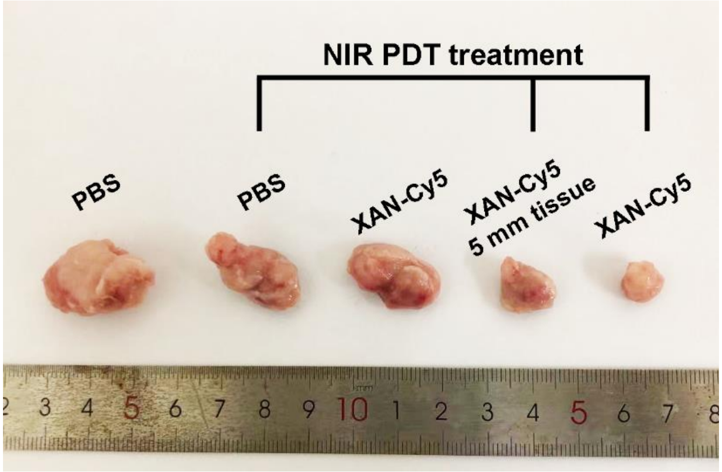

Figure S33. *Ex vivo* images of tumors in different treatment groups.

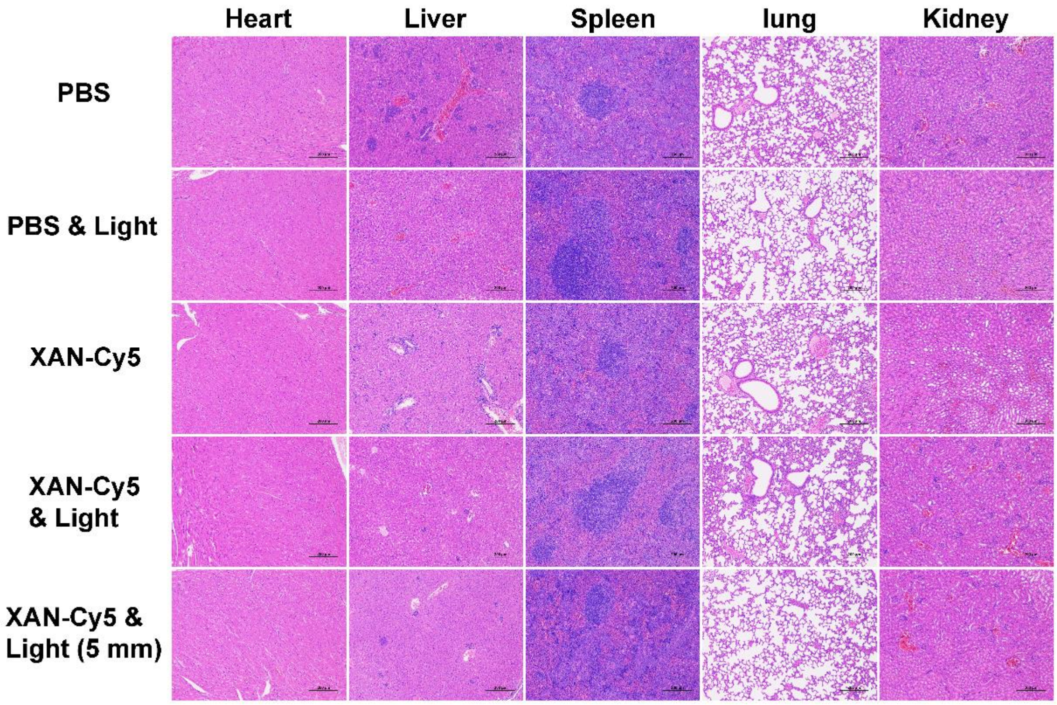

Figure S34. Typical images of H&E-stained heart, liver, spleen, lung, and kidney slices from mice 14 days post different treatments, scale bar = 200 μm.

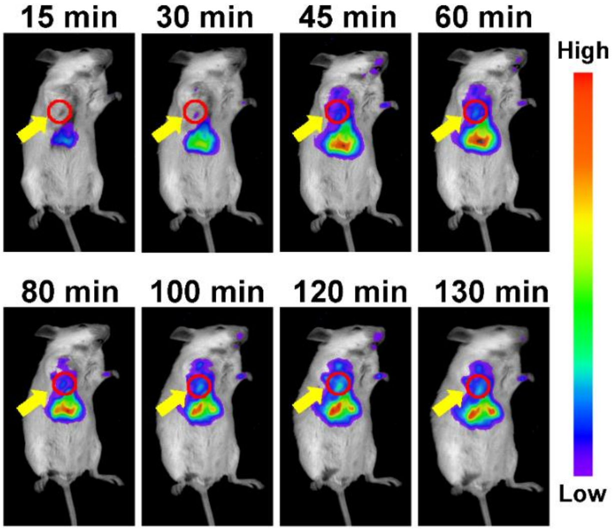

Figure S35. *In vivo* NIR tumor-targeting images of **XAN-Cy5.5** by *i.v.* injection.

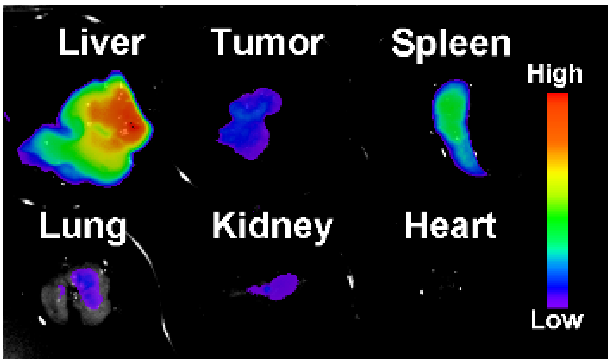

Figure S36. *Ex vivo* imaging of major organs, including heart, liver, spleen, lung, kidney and tumor at 120 min post *i.v.* injection of **XAN-Cy5.5**.

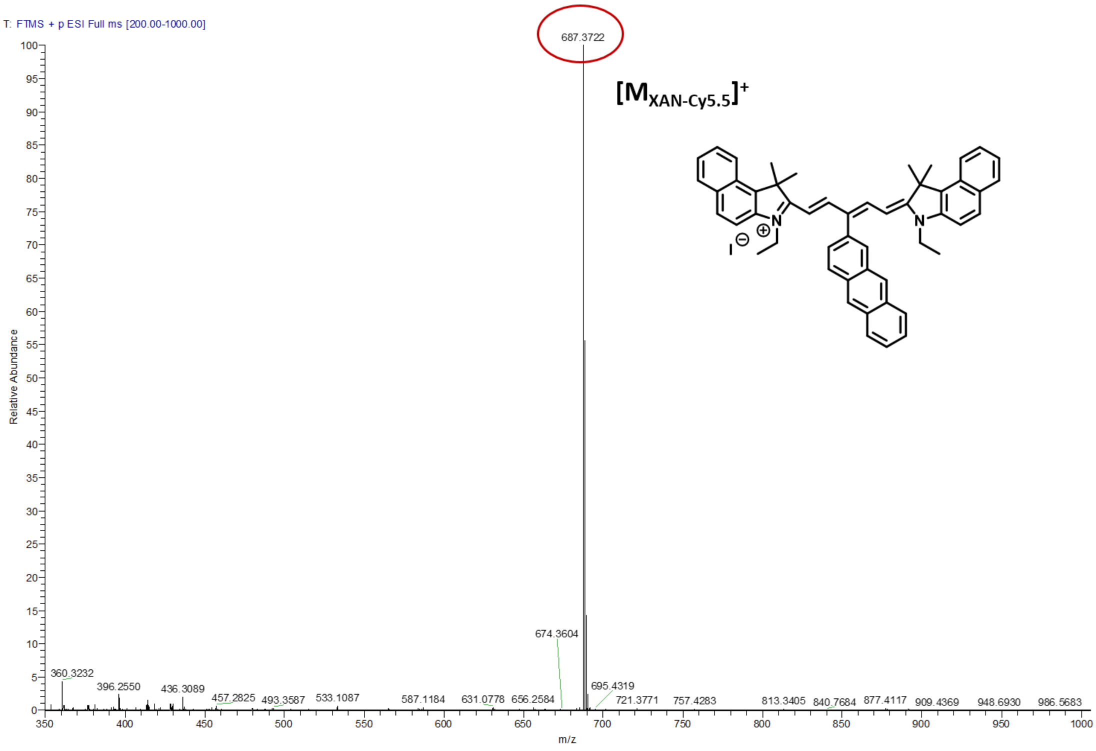

Figure S37. ESI-HRMS spectrum of **XAN-Cy5.5**.

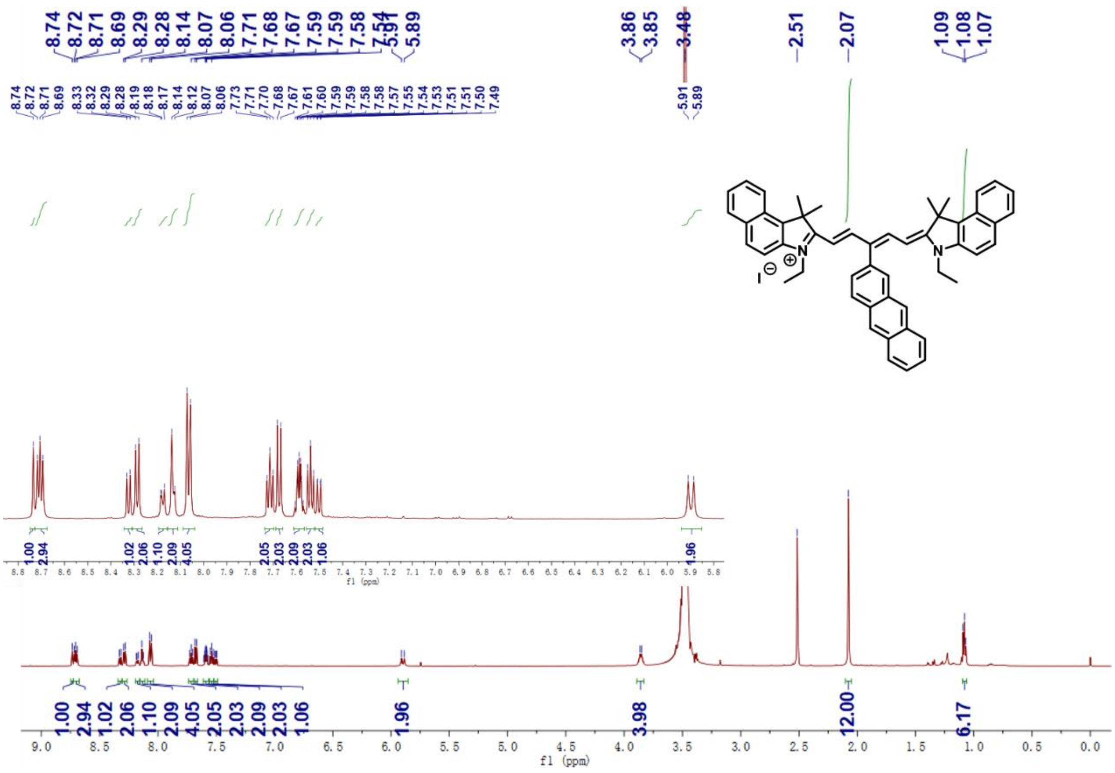

Figure S38. ^{1}H NMR spectrum of **XAN-Cy5.5**.

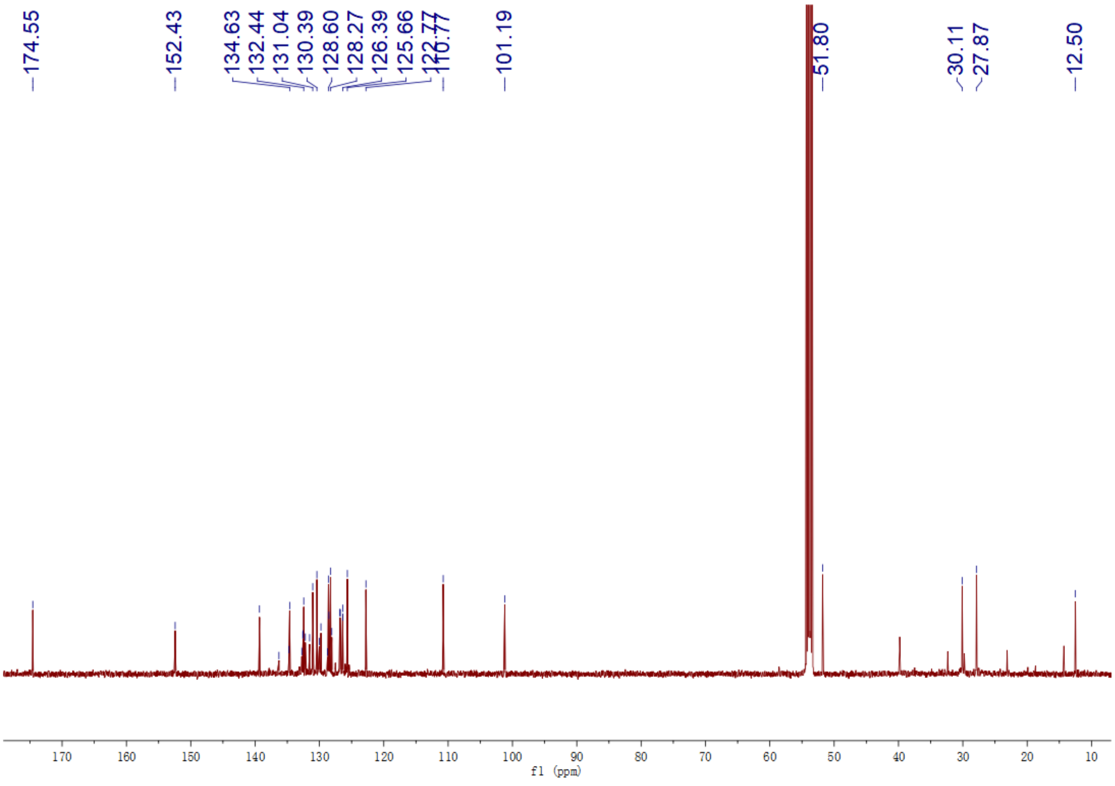

Figure S39. ^{13}C NMR spectrum of **XAN-Cy5.5**.

References

1. Zhao, Y.; Truhlar, D. G., The M06 suite of density functionals for main group thermochemistry, thermochemical kinetics, noncovalent interactions, excited states, and transition elements: two new functionals and systematic testing of four M06-class functionals and 12 other functionals. *Theor. Chem. Acc.* 2008, 120, 215-241.
2. Marenich, A. V.; Cramer, C. J.; Truhlar, D. G., Universal Solvation Model Based on Solute Electron Density and on a Continuum Model of the Solvent Defined by the Bulk Dielectric Constant and Atomic Surface Tensions. *The Journal of Physical Chemistry B* 2009, 113, 6378-6396.
3. Frisch, M. J.; Trucks, G. W.; Schlegel, H. B.; Scuseria, G. E.; Robb, M. A.; Cheeseman, J. R.; Scalmani, G.; Barone, V.; Petersson, G. A.; Nakatsuji, H.; Li, X.; Caricato, M.; Marenich, A. V.; Bloino, J.; Janesko, B. G.; Gomperts, R.; Mennucci, B.; Hratchian, H. P.; Ortiz, J. V.; Izmaylov, A. F.; Sonnenberg, J. L.; Williams; Ding, F.; Lipparini, F.; Egidi, F.; Goings, J.; Peng, B.; Petrone, A.; Henderson, T.; Ranasinghe, D.; Zakrzewski, V. G.; Gao, J.; Rega, N.; Zheng, G.; Liang, W.; Hada, M.; Ehara, M.; Toyota, K.; Fukuda, R.; Hasegawa, J.; Ishida, M.; Nakajima, T.; Honda, Y.; Kitao, O.; Nakai, H.; Vreven, T.; Throssell, K.; Montgomery Jr., J. A.; Peralta, J. E.; Ogliaro, F.; Bearpark, M. J.; Heyd, J. J.; Brothers, E. N.; Kudin, K. N.; Staroverov, V. N.; Keith, T. A.; Kobayashi, R.; Normand, J.; Raghavachari, K.; Rendell, A. P.; Burant, J. C.; Iyengar, S. S.; Tomasi, J.; Cossi, M.; Millam, J. M.; Klene, M.; Adamo, C.; Cammi, R.; Ochterski, J. W.; Martin, R. L.; Morokuma, K.; Farkas, O.; Foresman, J. B.; Fox, D. J. *Gaussian 16 Rev. A.03*, Wallingford, CT, 2016.
4. Neese, F., The ORCA program system. *Wiley Interdisciplinary Reviews: Computational Molecular Science* 2012, 2, 73-78.
5. Neese, F., Software update: the ORCA program system, version 4.0. *Wiley Interdisciplinary Reviews: Computational Molecular Science* 2018, 8, e1327.
6. Niu, Y.; Li, W.; Peng, Q.; Geng, H.; Yi, Y.; Wang, L.; Nan, G.; Wang, D.; Shuai, Z., MOlecular MAterials Property Prediction Package (MOMAP) 1.0: a software package for predicting the luminescent properties and mobility of organic functional materials. *Molecular Physics* 2018, 116, 1078-1090.
7. Huang, H.; Long, S.; Li, M.; Gao, F.; Du, J.; Fan, J.; Peng, X., Bromo-pentamethine as mitochondria-targeted photosensitizers for cancer cell apoptosis with high efficiency. *Dyes and Pigments* 2018, 149, 633-638.
8. Zhao, X.; Yao, Q.; Long, S.; Chi, W.; Yang, Y.; Tan, D.; Liu, X.; Huang, H.; Sun, W.; Du, J.; Fan, J.; Peng, X., An Approach to Developing Cyanines with Simultaneous Intersystem Crossing Enhancement and Excited-State Lifetime Elongation for Photodynamic Antitumor Metastasis. *Journal of the American Chemical Society* 2021, 143, 12345-12354.
9. Tian, R.; Wang, C.; Chi, W.; Fan, J.; Du, J.; Long, S.; Guo, L.; Liu, X.; Peng, X., Emerging Design Principle of Near-Infrared Upconversion Sensitizer Based on Mitochondria-Targeted Organic Dye for Enhanced Photodynamic Therapy. *Chemistry – A European Journal* 2021, 27, 16707-16715.
